# Shaping light in 3D for direct asymmetric photochemistry: a 1000-fold enantioselectivity enhancement

**DOI:** 10.1038/s41467-026-77096-w

**Published:** 2026-09-04

**Authors:** Andrés Ordóñez, Daniel García González, Patricia Vindel-Zandbergen, David Ayuso

**Affiliations:** 1 Department of Physics, Freie Universität Berlin, Berlin, Germany; 2 Department of Physics, Imperial College London, London, UK; 3 Department of Chemistry, Queen Mary University of London, London, UK; 4 Department of Chemistry, New York University, New York, NY USA; 5 Max-Born-Institut, Berlin, Germany; 6 Department of Chemistry, Molecular Sciences Research Hub, Imperial College London, London, UK

**Keywords:** Nonlinear optics, Nonlinear optics, Photochemistry

## Abstract

Shaping electronic motion with tailored light enables unprecedented control over matter: from realising topological states in quantum materials to steering chemical reactions along specific pathways. Yet, despite the vital roles of molecular chirality across nature, standard chiral light can barely influence the handedness of a chemical reaction, which is determined by the three-dimensional geometry of reactants and catalysts. Here we show how, by twisting light’s Lissajous figure into a three-dimensional chiral structure, we can selectively excite one molecular enantiomer over its mirror image to trigger direct asymmetric photochemistry with an unprecedented degree of enantioselectivity. Our ab-initio simulations reveal ~30% enantioselectivity in the electronic excitation of the chiral molecule carvone—a three-orders-of-magnitude enhancement over current optical methods using circularly polarised light (~0.01%). Such control over the primary step of a photochemical reaction opens up a highly efficient route for manipulating molecular chirality exclusively with light. Thus, circumventing the current need for chiral catalysts or enantiopure reactants, and bringing direct asymmetric photochemistry into the realm of applications.

## Abstract (web summary)

Recent progress in light shaping techniques enable control of chemical reaction pathways, yet chirality remains difficult to manipulate. Here, the authors demonstrate that tailoring the chirality of the Lissajous figure of the light electric field can achieve remarkable enantioselectivity of electronic excitations, opening new avenues towards coherent quantum control of molecular chirality.

## Introduction

We rely on different systems having different energy spectra not only to distinguish them, but also to selectively excite them, with far-reaching applications such as fluorescence microscopy^1–3^ or magnetic resonance imaging^4,5^. However, when two systems differ only by a mirror reflection, like the opposite enantiomers of a chiral molecule, selective excitation requires exploiting light properties beyond the wavelength, such as the helicity. This poses a fundamental challenge that continues to drive scientific efforts motivated by the ubiquity of molecular chirality. Beyond their well established roles in the pharmaceutical and agrochemical sectors, small chiral molecules are also emerging as disease biomarkers^6^ and becoming essential in nanotechnology^7^ for developing molecular motors^8^ and switches^9^, spintronic devices^10^, and self-assembling structures^11^.

Asymmetric synthesis is a cornerstone of modern chemistry enabling the selective production of one enantiomer over its mirror image. Current approaches rely on molecule-molecule interactions to drive high enantioselectivity, for example with chiral catalysts^12,13^ that make the potential energy landscapes enantioselective. This is also the case in photochemical applications^14,15^, where the role of light is limited to providing energy to trigger the reaction, with the enantioselectivity coming from a chiral molecular environment.

Light’s chirality as a source of enantioselectivity—the essence of direct asymmetric photochemistry—is typically limited below 0.1% ^16–19^. In the case of asymmetric synthesis, illumination with circularly polarised light (CPL) of a racemic mixture (partial photoresolution^16,20^) leads to virtually racemic products with vanishingly small enantiomeric excess (typically *e*. *e*. < 0.1%). Similarly, enantiopurification of racemic mixtures via photolysis requires destroying virtually the entire sample (99.99%) to achieve significant enantiomeric excess (*e*. *e*. ~ 35%)^18,21^. This weak enantioselectivity has long been a major hurdle for applications of asymmetric photochemistry, preventing them from being direct.

The weak chiral light-matter coupling can be traced back to the unbalanced interplay between (strong) electric-dipole and (weak) magnetic-dipole interactions^22^. Superchiral light^23,24^ can balance this interference by weakening the electric-dipole response over tiny spatial regions (~10 nm), thus suppressing the overall yield and compromising synthetic applications. Balance can also occur in exceptional cases due to electric-dipole forbidden transitions^25,26^. In contrast, novel approaches relying exclusively on electric-dipole interactions enable enhanced chiral sensitivity without suppressing the overall response^27^. These include chiral sum-frequency generation^28^, Coulomb explosion imaging^29,30^, photoelectron circular dichroism^31^, as well as enantioselective detection^32^ and manipulation via microwave rotational excitation^33,34^, which could lead to enantioseparation^35^. However, despite major advances in light sources^36^, the possibility of efficiently controlling the chirality of photochemical reactions exclusively with light has so far remained elusive. Indeed, while *e*. *e*. ~30% has been achieved in the self-assembly of nanoparticles^11,37^, direct asymmetric photochemistry involving small- to medium-size organic molecules has remained barely enantioselective.

Here we show how, by twisting the Lissajous figure that light’s electric field vector traces in time into a 3D chiral structure^38^, we can selectively excite one molecular enantiomer over its mirror image with ~30% selectivity—over three orders of magnitude higher than what is possible with CPL. In contrast with CPL, such chiral light does not rely on the imbalanced interplay between electric- and magnetic-dipole transitions. Instead, it can realise a coherent quantum control^35,39–49^ mechanism through interference between two balanced electric-dipole quantum pathways, thus opening up a highly enantioselective route to direct asymmetric photochemistry: enantioselective molecule-molecule interactions, e.g. with chiral catalysts, are not needed.

## Results

**Fig. 1:**
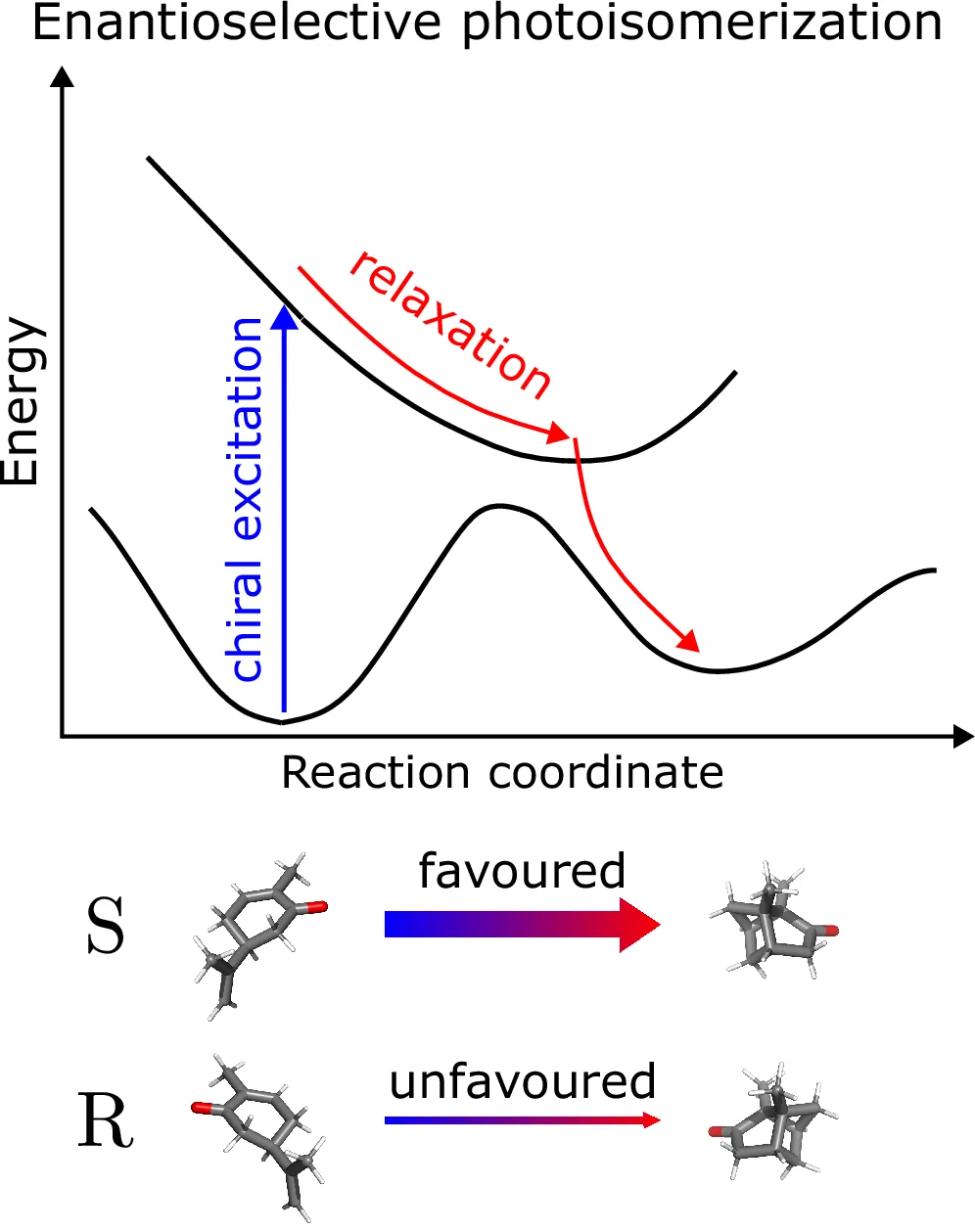
Direct asymmetric photochemistry. Chiral light drives an electronic excitation in a chiral molecule, which then relaxes to a new equilibrium geometry. The potential energy surfaces determining the course of the relaxation process are the same in both enantiomers. Chiral light preferentially excites one of the two enantiomers, thus favouring its photoisomerisation. In carvone, electronic excitation triggers isomerisation into carvonecamphor. Enantioselective excitation favours the production of e.g. S carvonecamphor.

We demonstrate our proposal by modelling the multiphoton interaction of our tailored light and spatially extended samples of randomly oriented carvone molecules in racemic mixtures (50% R - 50% S). Carvone is a garden-variety chiral molecule that played a central role in the early days of photochemistry^50–55^: photoexcitation triggers its isomerisation into carvonecamphor^52–57^, a widely studied $\left[2+2\right]$ photocycloaddition^58–60^, see Fig. 1. Once photoexcited, both carvone enantiomers are equally likely to isomerise into their respective enantiomer of carvonecamphor, as the energetics of the mirror-imaged potential energy surfaces are identical. Thus, enantioselective photoexcitation drives enantioselective formation of products. However, when the chemical reaction is initiated by CPL, the weak selectivity in the excitation step limits the enantioselectivity to ~0.01% ^61^.

Enantioselective population transfer within the electric-dipole approximation in randomly oriented samples requires interference between two quantum pathways, one involving an odd number of photons and the other an even number of photons^62^. Indeed, such an interference term contains an odd number of electric dipole transitions (**d**_1_ ⋅ **E**_1_)…(**d**_2*n*+1_ ⋅ **E**_2*n*+1_) and thus has opposite signs for opposite enantiomers (${{{\bf{d}}}}_{i}^{S}=-{{{\bf{d}}}}_{i}^{R}$). Moreover, the Lissajous figure drawn by the total electric field must be chiral, which requires non-coplanar polarisations and can be achieved by having at least two frequencies propagating in different directions.

In the simplest case, namely 1-vs-2-photon interference, one drives the 2-photon pathway with frequencies *ω*_1_ and *ω*_2_, and the 1-photon pathway with frequency *ω*_3_ = *ω*_1_ + *ω*_2_. One can show that the difference Δ*P* ≡ *P*_*R*_ − *P*_*S*_ between the excited state population in *R* and *S* enantiomers is given by (see ‘Methods’)

1 $$\Delta P({{\bf{r}}})=\Re \{{g}^{(3)}{h}^{(3)}({{{\bf{r}}}}_{0})\,{e}^{i\Delta {{\bf{k}}}\cdot ({{\bf{r}}}-{{{\bf{r}}}}_{0})}\},$$

where *g*^(3)^ and ${h}^{(3)}\propto [{\hat{{{\bf{e}}}}}_{3}^{*}\cdot ({\hat{{{\bf{e}}}}}_{1}\times {\hat{{{\bf{e}}}}}_{2})]$ are the relevant molecular and field pseudoscalars, which have opposite signs for molecules/fields of opposite handedness, ${\hat{{{\bf{e}}}}}_{j}$ is the polarisation of frequency *ω*_*j*_, *j* = 1, 2, 3; **r**_0_ is an arbitrary choice of origin, and Δ**k** ≡ **k**_1_ + **k**_2_ − **k**_3_ is the mismatch between the wavevectors **k**_*j*_. Equation (1) shows that the enantioselectivity Δ*P* oscillates in space with a period Λ ≡ 2π/∣Δ**k**∣ and an amplitude proportional to $[{\hat{{{\bf{e}}}}}_{3}^{*}\cdot ({\hat{{{\bf{e}}}}}_{1}\times {\hat{{{\bf{e}}}}}_{2})]$. That is, Δ*P* is maximised by polarisations perpendicular to each other. However, this requires having at least one frequency (e.g. *ω*_1_) propagating perpendicular to the other two, leading to a spatial period Λ ~ *λ*_1_ ≡ 2π/∣**k**_1_∣. Crucially, averaging over interaction regions of size similar or bigger than Λ washes out the enantioselectivity. While this does not prevent experiments using rotational transitions in the microwave domain (*λ*_1_ ~ 10 cm)^33,34,42,46,49,63^, it poses a serious challenge for direct asymmetric photochemistry (*λ*_1_ ~ 1 μm). This challenge can be addressed via a 2-vs-3-photon interference that supports Δ**k** ≠ **0**, thus suppressing the spatial oscillations of Δ*P*.

**Fig. 2:**
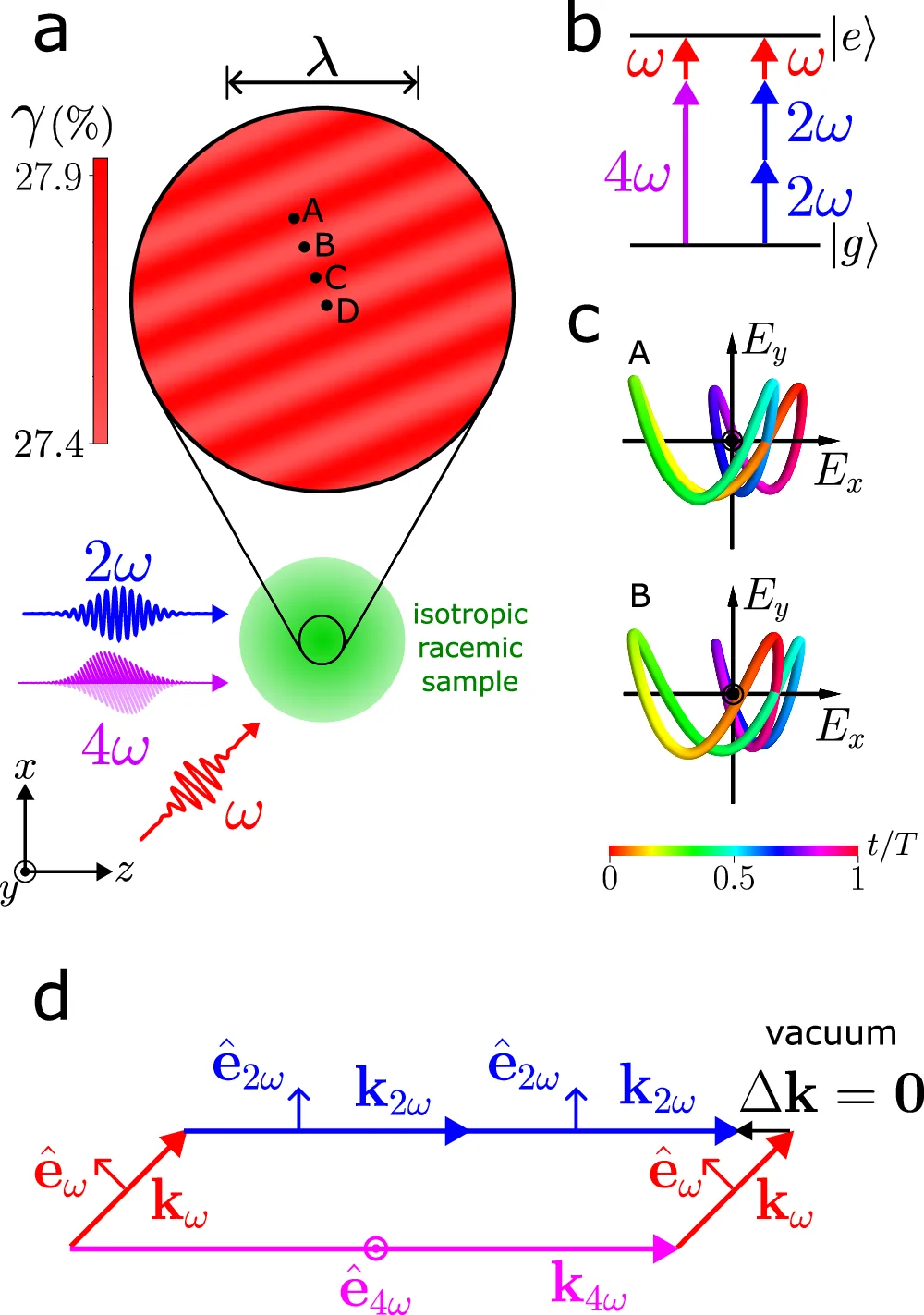
Multiphoton chiral excitation. **a** Enantioselectivity *γ* [Eq. (2)] as a function of position in a racemic, randomly oriented sample of carvone molecules using chiral 3D-tailored light with frequencies *ω*, 2*ω*, and 4*ω*. **b** Frequencies (5*ω* = 3.89 eV) and peak intensities (*I*_*ω*_ = 7.3 × 10^11^ W/cm^2^, *I*_2*ω*_ = 9.4 × 10^11^ W/cm^2^, *I*_4*ω*_ = 1.6 × 10^10^ W/cm^2^) are set to balance the weights of the two interfering pathways from the ground to the first electronic excited state, realising a 2- vs 3-photon coherent control scheme. The relative phase Δ*ϕ* = −0.186 rad between the frequencies 2*ω* and 4*ω* was set to maximise *γ* (see Fig. 3). All frequencies have a pulse duration of 22 fs (full-width half-maximum of intensity). **c** Polarisations (${\hat{{{\bf{e}}}}}_{\omega }=(\hat{{{\bf{x}}}}-\hat{{{\bf{z}}}})/\sqrt{2}$, ${\hat{{{\bf{e}}}}}_{2\omega }=\hat{{{\bf{x}}}}$, ${\hat{{{\bf{e}}}}}_{4\omega }=\hat{{{\bf{y}}}}$) and propagation directions (${\hat{{{\bf{k}}}}}_{\omega }=(\hat{{{\bf{x}}}}+\hat{{{\bf{z}}}})/\sqrt{2}$, ${\hat{{{\bf{k}}}}}_{2\omega }={\hat{{{\bf{k}}}}}_{4\omega }=\hat{{{\bf{z}}}}$) yield chiral Lissajous figures that change along the direction ${\hat{{{\bf{k}}}}}_{\omega }-{\hat{{{\bf{k}}}}}_{2\omega }$ on the scale of the fundamental wavelength *λ* ≡ 2*πc*/*ω* = 1.56 μm, but without reversing chirality. Lissajous figures at points A and B in **a** are different, but are not mirror images of each other. Lissajous figures at C and D are equal to those at A and B, respectively, rotated by 180^∘^ around $\hat{{{\bf{y}}}}$. The ABCD pattern repeats indefinitely. *T* ≡ 2*π*/*ω*. **d** Wavevector mismatch. It yields *Λ*/*λ* → *∞* in the absence of dispersion and *Λ*/*λ* = 33 assuming the dispersion of liquid water (*λ* = 1.2 μm) [see Eq. (3)].

Consider the interference between the 4*ω* + *ω* and 2*ω* + 2*ω* + *ω* quantum pathways shown in Fig. 2b, with 5*ω* = 3.89 eV resonant with the first electronic excitation (*λ* = 2π*c*/*ω* = 1.59 μm). Since both pathways involve absorption of one *ω* photon, their interference only depends on the relative phase between the 2*ω* and 4*ω* fields. That is, in contrast to the 1-vs-2-photon case, now the phase of the *ω* field cancels out. This gives us the opportunity to eliminate the spatial oscillations of enantioselectivity by having the 2*ω* and 4*ω* frequencies phase locked in the same beam, propagating at a 45^∘^ angle with respect to the *ω* beam, as shown in Fig. 2a.

We calculated the probability *P*_*S*/*R*_ of exciting randomly oriented *S*/*R* carvone molecules at their equilibrium geometry using state-of-the-art computational modelling (see ‘Methods’). Our simulations show that, for this 2-vs-3-photon interferometric approach, the normalised enantioselectivity

2 $$\gamma \equiv \frac{{P}_{R}-{P}_{S}}{\frac{1}{2}({P}_{R}+{P}_{S})}$$

can reach *γ* ≃ 28%—a 1000-fold enhancement with respect to what is possible with CPL^61^—across the whole interaction region (see Fig. 2a). The small fluctuations (<1%) of *γ* in Fig. 2 can be attributed to small contributions from other excitation pathways.

We can understand these results by analysing how the field polarisation changes in space, see Fig. 2c. The Lissajous figures follow the periodic pattern A, B, C, D. Lissajous figures at positions A and C are identical up to a 180^∘^ rotation and thus have the same chirality, leading to *γ*(A) = *γ*(C). Likewise, *γ*(B) = *γ*(D). The weak oscillations in the value of *γ*, e.g. *γ*(A) ≠ *γ*(B), result from the non-trivial modulation of the field’s polarisation: the Lissajous figures at positions A and B are approximately related to each other by time reversal, but are not related to each other by rotation or reflection. Indeed, the field never reverses chirality: one cannot find two spatial positions with Lissajous figures that are mirror images of each other. This is in stark contrast with the 1-vs-2-photon case, where the Lissajous figure reverses handedness on sub-wavelength scale, leading to sub-wavelength changes in the sign of *γ*. Furthermore, while using three linear polarisations perpendicular to each other maximises ∣*γ*∣ in the 1-vs-2-photon case, it leads to *γ* = 0 in the 2-vs-3-photon case. Indeed, the Lissajous figures in the latter case become achiral if we change the angle between the two beams to 90^∘^.

**Fig. 3:**
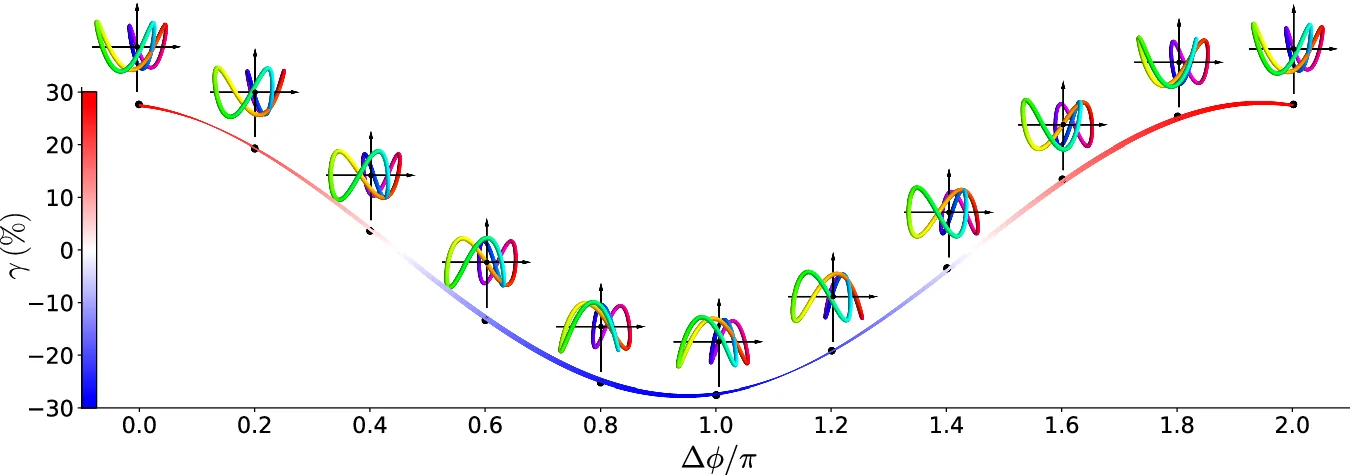
Coherent control over enantioselectivity. Enantioselectivity *γ* [Eq. (2)] as a function of the relative phase Δ*ϕ* between frequencies 2*ω* and 4*ω* for the scheme shown in Fig. 2, along with the corresponding Lissajous figures at point A. Variations of the enantioselectivity as a function of position in the interaction region fall within the thickness of the curve. The dots show calculations and the solid line a fit to ${\gamma }_{0}\cos (\Delta \phi+\delta )$ with *γ*_0_ = 27.9 % and *δ* = 0.186 rad. For any Δ*ϕ*, the position dependence of *γ* is similar to that shown in Fig. 2a, scaled to the value of *γ* shown here.

We can control the field’s chirality across the interaction region, and thus the degree of enantioselectivity, via the relative phase Δ*ϕ* between 4*ω* and 2*ω* frequencies. As shown in Fig. 3, shifting Δ*ϕ* by π reverses the field’s chirality everywhere in space, thus changing the sign of *γ*, i.e. changing which enantiomer is preferentially excited. Thus, our calculations show that by careful design of the excitation scheme, the enantioselectivity can be coherently controlled and kept virtually constant over distances much greater than the wavelength, spanning macroscopic interaction regions.

**Fig. 4:**
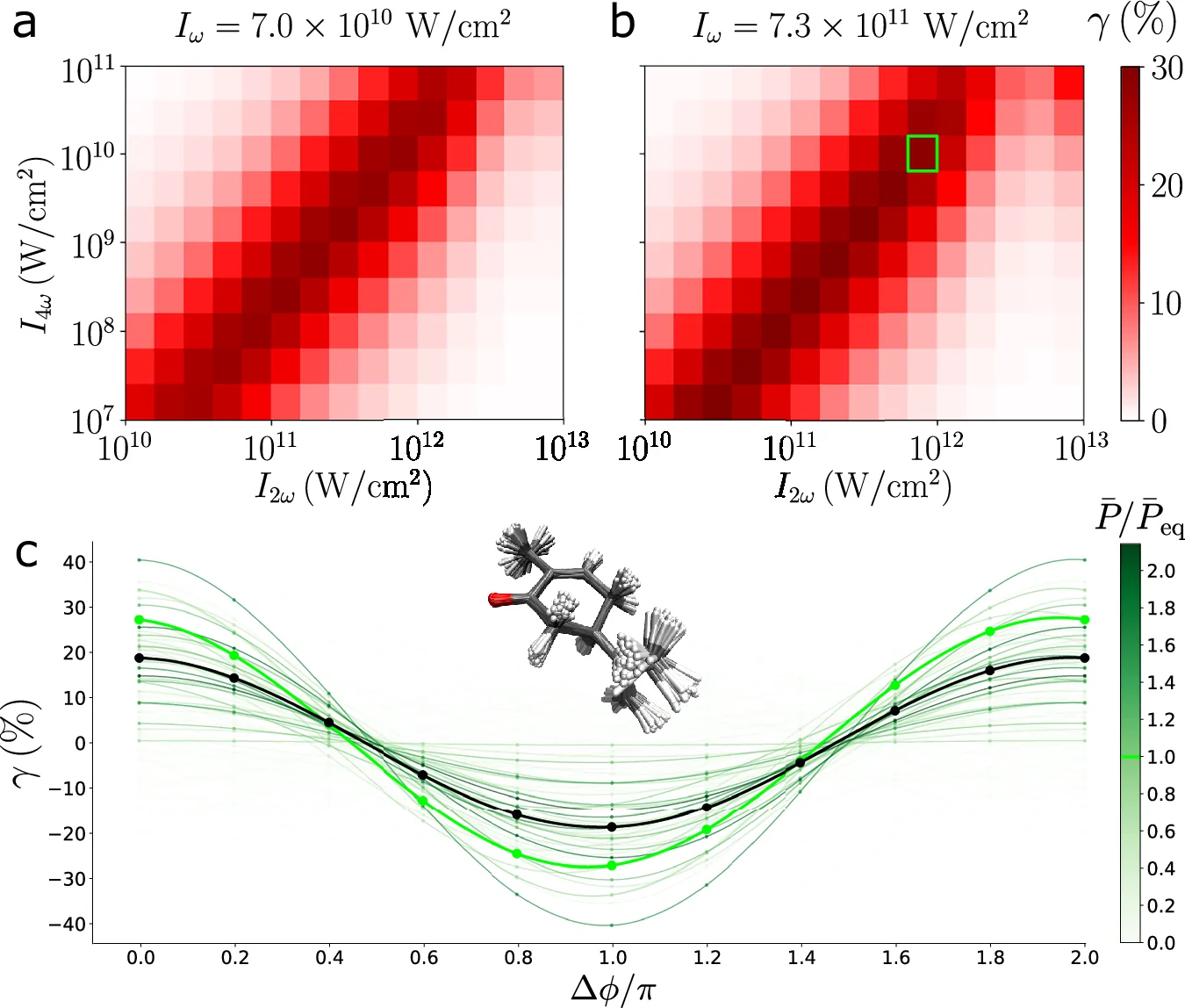
Stability of the coherent control scheme. **a** Enantioselectivity *γ*(Δ*ϕ* = 0) [Eq. (2) and Fig. 3] as a function of variations in the field intensities *I*_4*ω*_ and *I*_2*ω*_ for *I*_*ω*_ = 7.0 × 10^10^ W/cm^2^ and **b** *I*_*ω*_ = 7.3 × 10^11^ W/cm^2^. The green rectangle indicates the intensities used in Figs. 2 and 3. **c** Coherent control of enantioselectivity *γ*(Δ*ϕ*) for 100 different molecular geometries sampled from the Wigner distribution of the vibrational ground state, using the field parameters in Fig. 2. Each curve corresponds to a sampled geometry (see inset for the *R* enantiomer). Its colour indicates the excited state population $\bar{P}\equiv ({P}_{R}+{P}_{S})/2$, which varies by less than 2% as a function of Δ*ϕ*, normalised by that of the equilibrium geometry ${\bar{P}}_{{{\rm{eq}}}}$. The dots show calculations and the solid line a cubic interpolation. The thick black curve shows the enantioselectivity calculated adding populations over all sampled geometries. The thick green curve shows the results for the equilibrium geometry (see Fig. 3).

To address the robustness of our approach with respect to variations in the intensity of the driving fields, we calculated the enantioselectivity *γ* at Δ*ϕ* = 0 for a wide range of intensity combinations, as shown in Fig. 4a, b. For a fixed value of *I*_*ω*_, one can see that high enantioselectivity requires a balance between *I*_2*ω*_ and *I*_4*ω*_, which control the weight of each pathway (see Fig. 2b). As can be seen in Fig. 4a, b, this balance can be maintained over a broad range of intensities. Moreover, since one *ω* photon enters each pathway, the balance between the two pathways is largely independent of *I*_*ω*_, as can be seen from the similarity between Fig. 4a and b, which correspond to values of *I*_*ω*_ differing by over an order of magnitude.

To address robustness with respect to molecular parameters, we calculated the coherent control curve *γ*(Δ*ϕ*) for 100 molecular geometries randomly sampled from a Wigner distribution of the vibrational ground state of carvone (see Fig. 4c), while keeping all field parameters fixed to their values in Fig. 3. Of the 100 sampled geometries, 45 remain in near 5*ω* resonance with the applied field and are thus significantly excited, as indicated by the colour scale in Fig. 4c. Although the amplitude of the individual *γ*(Δ*ϕ*) curves is very sensitive to their molecular geometry, their phase is largely preserved, and thus the enantioselectivity survives ensemble averaging. Specifically, we still obtain a high total enantioselectivity of *γ* ≈ 19% (see thick black curve in Fig. 4c) when we compute it using ${P}_{S/R}={\sum}_{i}{P}_{S/R}^{(i)}$, where ${P}_{S/R}^{(i)}$ is the population for the *i*-th geometry in the *R*/*S* enantiomer and the sum runs over all configurations. This shows that the enantioselective response remains robust under averaging over the ground-state nuclear distribution, reflecting the fact that all the molecular geometries share the same underlying molecular chirality. We have not attempted an optimisation of the total enantioselectivity, thus *γ* ≈ 19% is a lower bound for the optimal total enantioselectivity.

Our analytical model (see ‘Methods’) shows that, for the 2-vs-3-photon interference, the enantioselectivity is determined by

3 $$\Delta P({{\bf{r}}})=\Re \{{{{\bf{g}}}}^{(5)}\cdot {{{\bf{h}}}}^{(5)}({{{\bf{r}}}}_{0})\,{e}^{i\Delta {{\bf{k}}}\cdot ({{\bf{r}}}-{{{\bf{r}}}}_{0})}\},$$

where, in contrast to the 1-vs-2-photon interference [see Eq. (1)], Δ**k** = **0** in the absence of dispersion (see Fig. 2c), leading to Λ → ∞, i.e. constant enantioselectivity across the interaction region. For the dispersion of liquid water, Λ = 2π/∣Δ**k**∣ = 33*λ*, where *λ* = 2π*c*/*nω* = 1.2 μm. **g**^(5)^ and **h**^(5)^ are molecular and field pseudovectors.

**h**^(5)^ encodes the field’s handedness relevant for this excitation scheme,

4 $${{{\bf{h}}}}^{\left(5\right)}\equiv \left(\begin{array}{c}\left[{{{\bf{E}}}}_{2\omega }\cdot ({{{\bf{E}}}}_{\omega }\times {{{\bf{E}}}}_{\omega }^{*})\right]({{{\bf{E}}}}_{2\omega }\cdot {{{\bf{E}}}}_{4\omega }^{*})\\ \left[{{{\bf{E}}}}_{2\omega }\cdot ({{{\bf{E}}}}_{\omega }\times {{{\bf{E}}}}_{4\omega }^{*})\right]({{{\bf{E}}}}_{2\omega }\cdot {{{\bf{E}}}}_{\omega }^{*})\\ \left[{{{\bf{E}}}}_{2\omega }\cdot ({{{\bf{E}}}}_{\omega }^{*}\times {{{\bf{E}}}}_{4\omega }^{*})\right]({{{\bf{E}}}}_{2\omega }\cdot {{{\bf{E}}}}_{\omega })\end{array}\right),$$

where **E**_*jω*_, is the electric-field amplitude of frequency *jω* including polarisation and relative phase, see ‘Methods’. Equations (3) and (4) are valid for arbitrary polarisations of the three frequencies. Each component of ${{{\bf{h}}}}^{\left(5\right)}$ is more nuanced than *h*^(3)^ [see Eq. (1)], as they involve not only a triple product but also a dot product. Thus, for a component of ${{{\bf{h}}}}^{\left(5\right)}$ to be non-zero, not only three of the electric field vectors must be non-coplanar, the other two must be non-orthogonal. In particular, if the three frequencies are linearly polarised perpendicular to each other, all components of **h**^(5)^ vanish, leading to *γ* = 0, thus reflecting that the Lissajous figure becomes achiral.

Our analytical model reveals a fundamental consequence of the vectorial nature of ${{{\bf{g}}}}^{\left(5\right)}$ and ${{{\bf{h}}}}^{\left(5\right)}$ in Eq. (3). Because the orientation of ${{{\bf{g}}}}^{\left(5\right)}$ is molecule specific, so is the orientation of ${{{\bf{h}}}}^{\left(5\right)}$ that maximises *γ*. This means one can maximise *γ* by adjusting the orientation of **h**^(5)^ via the field polarisation to align it with **g**^(5)^. For instance, for the polarisations used in Fig. 2a, **h**^(5)^ ∝ (0, 1, 1). In contrast, choosing *ω*_1_ circularly polarised and *ω*_2_ and *ω*_3_ linearly polarised perpendicular to the plane of circular polarisation yields **h**^(5)^ ∝ (1, 0, 0), see also refs. ^48^. Alternatively, if in Fig. 2a we take *ω* circularly polarised, ${{{\bf{E}}}}_{2\omega }\propto \hat{{{\bf{y}}}}$ and ${{{\bf{E}}}}_{4\omega }\propto \hat{{{\bf{x}}}}$, we obtain **h**^(5)^ ∝ (0, − 1, 1). The merit of each of these polarisation combinations and their corresponding **h**^(5)^ vectors depends on the details of **g**^(5)^, which varies from molecule to molecule and remains to be systematically investigated. This is in contrast with the 1-vs-2-photon interference, where the scalar character of ${g}^{\left(3\right)}$ and ${h}^{\left(3\right)}$ means that the optimal polarisation combination is the same for all chiral molecules, up to relative phases.

## Discussion

Coherent control of electronic population in polyatomic molecules has been experimentally demonstrated in gas and liquid samples^64,65^. In particular, in the liquid phase, interference between simultaneously driven 2-photon pathways in the absence of intermediate resonances has proven to be a robust approach to controlling population transfer^66–77^. Our approach relies on tailoring a light field using three frequencies, which may be an intense IR source and two of its harmonics, to simultaneously drive 2- and 3-photon pathways in the absence of intermediate resonances, without resorting to excited wave packet dynamics, and requiring only a stable relative phase between the two co-propagating frequencies. This is well within reach of state-of-the-art field synthesis^78,79^, which can provide optical pulses carrying multiple frequencies spanning from the UV to the near-IR, with high intensities, and controlled relative phases, at kHz repetition rates^80–85^, and in non-collinear geometries^86,87^. Furthermore, the integration of liquid microjet technology in femtosecond laser setups^88–93^ facilitates multiphoton experiments by providing a continuously refreshed, thin sample that minimises optical scattering, dispersion, and thermal effects. For such thin samples, a large angle between the non-collinear beams does not significantly reduce the volume of the interaction region. Moreover, the sample can be recycled and pass multiple times through the interaction region.

There are several alternatives to probe enantioselective population transfer to an electronic state. If the excited state decays via isomerisation, as in carvone, it will favour the creation of one of the two enantiomers of the photoproduct. Thus, starting from a racemic sample, enantioselective excitation will lead to a non-racemic mixture, both for starting and product molecules^21,94^. This change can be monitored using standard enantiosensitive methods, either spectroscopically or chemically, as usually done in asymmetric catalysis^15,95^. Alternatively, if the excited state decays via fluorescence, one could start with an enantiopure sample and monitor how fluorescence changes upon reversal of the field chirality **h**^(5)^, as done with CPL or superchiral light^23^. Analogously, in the gas phase, instead of measuring fluorescence, one could also probe the excited state population via photoelectron spectroscopy^60,,96^, and monitor ionisation yields as a function of **h**^(5)^.

Direct asymmetric photochemistry, by avoiding reliance on chiral sensitisers, offers a general route to asymmetric synthesis that bypasses intricate intermolecular interactions and reaction mechanisms^15^. By shaping light in 3D, we can overcome the major hurdle that has so far prevented applications: the weak chiral light-matter coupling. Our results show a three-orders-of-magnitude enhancement over CPL^61^, which can be achieved with current laser technology^78,81^. In contrast with enantioselective population transfer in the microwave domain, our approach (i) directly addresses the primary step of a photochemical reaction: electronic excitation, (ii) requires neither rotationally cold molecules, nor quadruply-resonant excitation, nor narrow band excitation, nor long electronic coherence times, and (iii) is applicable to liquid samples^66–76^. Thus, we overcome several fundamental limitations of previous proposals^35,40–49^ and bring applications of direct asymmetric photochemistry within reach.

Our mathematical model reveals the mechanism underlying enantioselective population transfer by identifying the relevant molecular and field pseudoscalars in multiphoton excitation. It provides a recipe for controlling and maximising the degree of enantioselectivity via light shaping, according to the specific properties of the molecular sample and the capabilities of each laboratory. Furthermore, it reveals that the chiral light-matter coupling can take a vectorial rather than a scalar form, opening an opportunity for selectively addressing specific molecules in mixtures that goes beyond simply relying on differences in energy-level structure. Such mixtures are often unavoidable, as even pure samples typically exist as conformational mixtures. Similarly, in situations where the target state is nearly energy-degenerate with other excited states and the enantioselectivity may be reduced due to state averaging, the vast parameter space provided by the different possible polarisation combinations may offer a compromise between state selectivity and high enantioselectivity.

Our approach offers fundamentally new opportunities beyond asymmetric synthesis, such as rapid switching of enantioselectivity in time and space. This is a key capability required for the implementation of chiroptical molecular switches^9,97^, the practical implementation of which is currently thwarted by the weak enantioselectivity of CPL.

## Methods

### Simulations

In our simulations, we use the fixed nuclei approximation and the electric-dipole approximation. For a given set of electric field parameters, a fixed molecular orientation, and a fixed point in the interaction region, we expand the electronic wave function in terms of the unperturbed eigenstates and solve the time-dependent Schrödinger equation (TDSE) for the expansion coefficients. The population of the first excited state is then averaged over molecular orientations. The field intensities are set by requiring the weights of the two interfering pathways to be balanced. First we set *I*_2*ω*_ = 0, and adjust *I*_4*ω*_ and *I*_*ω*_ to reach *P*_1_ = *P*_target_, while keeping *I*_4*ω*_ < *I*_*ω*_. Then we set *I*_4*ω*_ = 0, keep *I*_*ω*_ as found in the previous step, and adjust *I*_2*ω*_ to obtain *P*_1_ = *P*_target_. Then we carry out the simulations using all the intensities found in the previous two steps. We use short Gaussian pulses ( ~ 20 fs full-width half-maximum in intensity) to limit population transfer (*P*_target_ = 10^−6^) and stay within the perturbative few-photon picture of Fig. 2b. We scan the excited state population as a function of the relative phase between the different frequencies of the electric field. This scan is used to reconstruct the population as a function of molecular position across the interaction region. This reconstruction is exact in the limit of long pulses with planar wave fronts.

We perform electronic structure calculations to compute the transition energies and dipole moments required to solve the TDSE. The structure of the most stable conformer of R carvone was taken from ref. ^61^ and reoptimized in the current work for consistency using Density Functional Theory (DFT)^98,99^ with the B3LYP^100,101^ functional and the 6-311G(d,p)^102^ basis set available in Gaussian 16^103^. Harmonic frequencies were computed at the same level and used to construct a Wigner distribution of the vibrational ground state, from which 100 molecular geometries were sampled to represent the initial nuclear wavefunction. The excitation energies and transition moments for the first 100 excited states were computed for each sampled geometry using time-dependent DFT with the CAM-B3LYP functional and the d-aug-cc-pVDZ basis set. The transition dipole moments between all excited states were evaluated using the Multiwfn software^104^. The performance of the CAM-B3LYP functional in the calculation of spectroscopic properties of chiral systems has been discussed in detail in refs. ^105–109^ and has been recently shown to predict circular dichroism spectra in good agreement with results obtained from highly accurate EOM-CCSD calculations^110^.

The integration over orientations is carried out using a 14-point Lebedev grid for *α* and *β*, and a trapezoidal rule with 9 equally spaced points in *γ*, for a total of 126 orientations, where *α*, *β*, *γ* are Euler angles in the *zyz* convention. Increasing the number of orientations to 286 (26-point Lebedev in *α* and *β* and 11 in *γ*) did not lead to any significant change.

### Relative phases and spatial dependence of the field

For an electric field consisting of several frequencies ${\{{\omega }_{j}\}}_{j=1}^{N}$, each of them a plane wave with amplitude *E*_*j*_, polarisation ${\hat{{{\bf{e}}}}}_{j}$, phase *ϕ*_*j*_, and propagating along the unit vector ${\hat{{{\bf{k}}}}}_{j}$ with speed *c*_*j*_, we can write

5 $$\tilde{{{\bf{E}}}}\left({{\bf{r}}},t;{\phi }_{1},{\phi }_{2},\ldots,{\phi }_{N}\right)\equiv \mathop{\sum }\limits_{j=1}^{N}{E}_{j}{e}^{-i\left({\omega }_{j}t+{\phi }_{j}-\frac{{\omega }_{j}}{{c}_{j}}{\hat{{{\bf{k}}}}}_{j}\cdot {{\bf{r}}}\right)}{\hat{{{\bf{e}}}}}_{j}.$$

Taking the frequency *ω*_1_ as a reference, one can show that a shift in position Δ**r** is equivalent to a simultaneous shift of time and of the phases of the rest of frequencies Δ*ϕ*_*j*_, *j* = 2, …*N*, according to

6 $$\tilde{{{\bf{E}}}}\left({{\bf{r}}}+\Delta {{\bf{r}}},t;{\phi }_{1},{\phi }_{2},\ldots,{\phi }_{N}\right)=\tilde{{{\bf{E}}}}\left({{\bf{r}}};t+\Delta t;{\phi }_{1},{\phi }_{2}+\Delta {\phi }_{2},\ldots,{\phi }_{N}+\Delta {\phi }_{N}\right),$$

where

7 $$\Delta t=-\frac{{\hat{{{\bf{k}}}}}_{1}\cdot \Delta {{\bf{r}}}}{{c}_{1}},\qquad \Delta {\phi }_{j}={\omega }_{j}\left(\frac{1}{{c}_{1}}{\hat{{{\bf{k}}}}}_{1}-\frac{1}{{c}_{j}}{\hat{{{\bf{k}}}}}_{j}\right)\cdot \Delta {{\bf{r}}}.$$

An overall time shift of the electric field does not change the final state populations. But the final state populations do depend on the phases *ϕ*_*j*_. Thus, knowing the populations for all *ϕ*_*j*_ at a given position **r** is enough to recover the populations at any other point **r** + Δ**r** via Eq. (6). This approximation is valid as long as the temporal envelopes do not change significantly upon the shift by Δ*t*. That is, if the pulse envelopes change on a time scale *τ*, Eq. (6) is valid provided ∣Δ**r**∣ ≪ *c*_1_*τ*. Taking *c*_1_ equal to the speed of light in vacuum and *τ* = 100 fs, this yields ∣Δ**r**∣ ≪ 30 μm. In our simulations, we use relatively short pulses *τ* ≈ 20 fs for the sake of reducing the computing time and avoiding nuclear dynamics, and thus our reconstruction is strictly valid only for ∣Δ**r**∣ ≪ 6 μm. However, our approach can be trivially extended to longer pulses and thus bigger ∣Δ**r**∣.

### Analytical model for 1- vs 2-photon isotropic chiral coherent control

Consider a chiral molecule interacting with a three-colour field given by $\tilde{{{\bf{E}}}}(t)={\sum }_{j=1}^{3}{\tilde{{{\bf{E}}}}}_{j}(t)$, where ${\tilde{{{\bf{E}}}}}_{j}(t)={{{\mathcal{E}}}}_{j}(t){{\rm{Re}}}\{{{{\bf{E}}}}_{j}{e}^{-i{\omega }_{j}t}\}$, ${{{\mathcal{E}}}}_{j}(t)$ describes a smooth temporal envelope and the complex vector **E**_*j*_ encodes the amplitude, polarisation, and phase of the frequency *ω*_*j*_. The frequencies are such that *ω*_1_ + *ω*_2_ = *ω*_3_ is resonant with the transition from the ground state $\left\vert g\right\rangle$ to the excited state $\left\vert e\right\rangle$. This leads to three sets of excitation pathways: a one photon pathway $\left\vert g\right\rangle \mathop{\to}^ {{\omega }_{3}}\left\vert e\right\rangle$, and two sets of two-photon pathways $\left\vert g\right\rangle {\to}^ {{\omega }_{2}}\left\vert n\right\rangle {\to}^ {{\omega }_{1}}\left\vert e\right\rangle$ and $\left\vert g\right\rangle {\to}^ {{\omega }_{1}}\left\vert n\right\rangle {\to}^ {{\omega }_{2}}\left\vert e\right\rangle$, that differ only by the photon ordering, and where $\left\vert n\right\rangle$ is an intermediate state that has to be summed over. The probability amplitude for state $\left\vert e\right\rangle$ after the interaction is given by

8 $${a}_{e}={a}_{e}^{\left({\omega }_{3}\right)}+\mathop{\sum }\limits_{n}\left({a}_{e,n}^{\left({\omega }_{1},{\omega }_{2}\right)}+{a}_{e,n}^{\left({\omega }_{2},{\omega }_{1}\right)}\right),$$

where perturbation theory yields

9 $${a}_{e}^{({\omega }_{3})}={A}^{\left({\omega }_{3}\right)}\left({{{\bf{d}}}}_{e,g}\cdot {{{\bf{E}}}}_{3}\right),$$

10 $${a}_{e,n}^{\left({\omega }_{1},{\omega }_{2}\right)}={A}_{n}^{\left({\omega }_{1},{\omega }_{2}\right)}\left({{{\bf{d}}}}_{e,n}\cdot {{{\bf{E}}}}_{1}\right)\left({{{\bf{d}}}}_{n,g}\cdot {{{\bf{E}}}}_{2}\right),$$

and ${a}_{e,n}^{\left({\omega }_{2},{\omega }_{1}\right)}$ is given by ${a}_{e,n}^{\left({\omega }_{1},{\omega }_{2}\right)}$ after replacing ${A}_{n}^{\left({\omega }_{1},{\omega }_{2}\right)}$ by ${A}_{n}^{\left({\omega }_{2},{\omega }_{1}\right)}$ and exchanging **E**_1_ and **E**_2_. Here ${{{\bf{d}}}}_{m,n}\equiv \left\langle m\right\vert {{\bf{d}}}\left\vert n\right\rangle$ is the electric dipole transition matrix element, and ${A}^{\left({\omega }_{3}\right)}$, ${A}_{n}^{\left({\omega }_{1}{\omega }_{2}\right)}$, and ${A}_{n}^{\left({\omega }_{2},{\omega }_{1}\right)}$ are coupling coefficients that depend on detunings and on the envelopes of the pulses. The orientation averaged population is given by *P*_*e*_ ≡ ∫ d*ϱ*∣*a*_*e*_∣^2^, where *ϱ* ≡ (*α*, *β*, *γ*) and $\int\,{{\rm{d}}}\varrho \equiv {(8{\pi }^{2})}^{-1}\int_{0}^{2\pi }{{\rm{d}}}\alpha \int_{0}^{\pi }{{\rm{d}}}\beta \sin \beta \int_{0}^{2\pi }{{\rm{d}}}\gamma$, which contains contributions from all three sets of pathways and their interferences. These can be classified according to the number of photons [i.e. electric-dipole products (**d** ⋅ **E**)] they contain. If they contain an even number of photons they are not enantiosensitive, whereas if they contain an odd number of photons they are enantiosensitive^62^. The latter result from the interference between the one photon pathway and either of the two-photon pathways. Such interferences contain expressions such as ${({{{\bf{d}}}}_{e,g}\cdot {{{\bf{E}}}}_{3})}^{*}({{{\bf{d}}}}_{e,n}\cdot {{{\bf{E}}}}_{1})({{{\bf{d}}}}_{n,g}\cdot {{{\bf{E}}}}_{2})$, involving three photons. The population in R and S enantiomers can thus be written as ${P}_{e}^{{{\rm{R}}}}={\overline{P}}_{e}+\frac{1}{2}\Delta {P}_{e}$ and ${P}_{e}^{{{\rm{S}}}}={\overline{P}}_{e}-\frac{1}{2}\Delta {P}_{e}$, where ${\overline{P}}_{e}=({P}_{e}^{{{\rm{R}}}}+{P}_{e}^{{{\rm{S}}}})/2$ is the non-enantiosensitive part and $\Delta {P}_{e}/2=({P}_{e}^{{{\rm{R}}}}-{P}_{e}^{{{\rm{S}}}})/2$ is the enantiosensitive part. The latter is given by

11 $$\frac{1}{2}\Delta {P}_{e}=2{{\rm{Re}}}\left\{\int{{\rm{d}}}\varrho \,{a}_{e}^{\left({\omega }_{3}\right)*}\mathop{\sum }\limits_{n}\left({a}_{e,n}^{\left({\omega }_{1},{\omega }_{2}\right)}+{a}_{e,n}^{\left({\omega }_{2},{\omega }_{1}\right)}\right)\right\}$$

12 $$=	\frac{1}{3}{{\rm{Re}}}\left\{{A}^{\left({\omega }_{3}\right)*}\mathop{\sum }\limits_{n}\left({A}_{n}^{\left({\omega }_{1},{\omega }_{2}\right)}-{A}_{n}^{\left({\omega }_{2},{\omega }_{1}\right)}\right)\left[{{{\bf{d}}}}_{e,g}^{\,{{\rm{R}}}}\cdot \left({{{\bf{d}}}}_{e,n}^{\,{{\rm{R}}}}\times {{{\bf{d}}}}_{n,g}^{\,{{\rm{R}}}}\right)\right]\right.\\ 	\left.\left[{{{\bf{E}}}}_{3}^{*}\cdot \left({{{\bf{E}}}}_{1}\times {{{\bf{E}}}}_{2}\right)\right]\right\},$$

where ${{{\bf{d}}}}_{m,n}^{\,X}$ are the dipoles of the X = R, S enantiomer, and ${{{\bf{d}}}}_{m,n}^{\,R}=-{{{\bf{d}}}}_{m,n}^{\,S}$. The triple products in Eq. (12) result from molecular orientation averaging^111^. The term $[{{{\bf{d}}}}_{e,g}^{\,{{\rm{R}}}}\cdot ({{{\bf{d}}}}_{e,n}^{\,{{\rm{R}}}}\times {{{\bf{d}}}}_{n,g}^{\,{{\rm{R}}}})]=-[{{{\bf{d}}}}_{e,g}^{\,{{\rm{S}}}}\cdot ({{{\bf{d}}}}_{e,n}^{\,{{\rm{S}}}}\times {{{\bf{d}}}}_{n,g}^{\,{{\rm{S}}}})]$ encodes the molecular chirality while $[{{{\bf{E}}}}_{3}^{*}\cdot ({{{\bf{E}}}}_{1}\times {{{\bf{E}}}}_{2})]$ encodes the chirality of the field. See Refs. ^38^ and ^112^ for examples in the context of nonlinear optics and vibrational dynamics, respectively. The factor $({A}_{n}^{\left({\omega }_{1},{\omega }_{2}\right)}-{A}_{n}^{\left({\omega }_{2},{\omega }_{1}\right)})$ in Eq. (12) is related to the opposite photon ordering of both contributions and the anti-commutativity of the triple product. In general, ${A}_{n}^{\left({\omega }_{1},{\omega }_{2}\right)}\ne {A}_{n}^{\left({\omega }_{2},{\omega }_{1}\right)}$, i.e. the energy structure favours a particular photon ordering. For illustrative purposes, we include here the coupling coefficients relevant for identical Gaussian envelopes ${{{\mathcal{E}}}}_{j}(t)=$ $\exp (-{t}^{2}/2{\tau }^{2})$

13 $${A}^{\left({\omega }_{3}\right)}=i\sqrt{\frac{\pi }{2}}\tau {e}^{-{\tau }^{2}{\Delta }_{eg}^{2}/2},$$

14 $${A}_{n}^{\left({\omega }_{1},{\omega }_{2}\right)}=\frac{i{\tau }^{2}}{4}{e}^{-{\tau }^{2}{\Delta }_{eg}^{2}/4}\left[2\sqrt{\pi }{D}_{+}(\xi )+i\pi {e}^{-{\xi }^{2}}\right],$$

where Δ_*eg*_ ≡ *ω*_*eg*_ − *ω*_3_, *ξ* ≡ (2Δ_*n*_ − Δ_*eg*_)*τ*/2, Δ_*n*_ ≡ *ω*_*n*0_ − *ω*_2_, and ${D}_{+}(x)\equiv {e}^{-{x}^{2}}\int_{0}^{x}{e}^{{t}^{2}}{{\rm{d}}}t$ is the Dawson function. The expression for ${A}_{n}^{\left({\omega }_{2},{\omega }_{1}\right)}$ can be obtained from ${A}_{n}^{\left({\omega }_{1},{\omega }_{2}\right)}$ by exchanging *ω*_1_ and *ω*_2_.

Equation (12) reduces to Eq. (1) after writing out the spatial oscillation of the field ${{{\bf{E}}}}_{j}({{\bf{r}}})={{{\bf{E}}}}_{j}({{{\bf{r}}}}_{0}){e}^{i{{{\bf{k}}}}_{j}\cdot ({{\bf{r}}}-{{{\bf{r}}}}_{0})}$, and defining *g*^(3)^ and *h*^(3)^ as

15 $${g}^{\left(3\right)}\equiv \frac{2}{3}{A}^{\left({\omega }_{3}\right)*}\mathop{\sum }\limits_{n}\left({A}_{n}^{\left({\omega }_{1},{\omega }_{2}\right)}-{A}_{n}^{\left({\omega }_{2},{\omega }_{1}\right)}\right)\left[{{{\bf{d}}}}_{e,g}^{\,{{\rm{R}}}}\cdot \left({{{\bf{d}}}}_{e,n}^{\,{{\rm{R}}}}\times {{{\bf{d}}}}_{n,g}^{\,{{\rm{R}}}}\right)\right],$$

16 $${h}^{(3)}\equiv {{{\bf{E}}}}_{3}^{*}\cdot \left({{{\bf{E}}}}_{1}\times {{{\bf{E}}}}_{2}\right).$$

Equation (16) provides a recipe to maximise ∣*h*^(3)^∣, and thus Δ*P*_*e*_, via polarisation shaping. One option is to have the three polarisations linear and perpendicular to each other. An equally effective choice is to take ${\hat{{{\bf{e}}}}}_{2}$ and ${\hat{{{\bf{e}}}}}_{3}$ circularly polarised and co-rotating, and ${\hat{{{\bf{e}}}}}_{1}$ linearly polarised perpendicular to the plane of circular polarisation [e.g. ${\hat{{{\bf{e}}}}}_{1}=\hat{{{\bf{z}}}}$, ${\hat{{{\bf{e}}}}}_{2}={\hat{{{\bf{e}}}}}_{3}=(\hat{{{\bf{x}}}}+i\hat{{{\bf{y}}}})/\sqrt{2}$]. Or ${\hat{{{\bf{e}}}}}_{1}$ and ${\hat{{{\bf{e}}}}}_{2}$ circularly polarised and counter-rotating, and ${\hat{{{\bf{e}}}}}_{3}$ linearly polarised and perpendicular to the plane of circular polarisation [e.g. ${\hat{{{\bf{e}}}}}_{1}=(\hat{{{\bf{x}}}}+i\hat{{{\bf{y}}}})/\sqrt{2}$, ${\hat{{{\bf{e}}}}}_{2}=(\hat{{{\bf{x}}}}-i\hat{{{\bf{y}}}})/\sqrt{2}$, ${\hat{{{\bf{e}}}}}_{3}=\hat{{{\bf{z}}}}$]. All of these choices maximise *h*^(3)^.

### Analytical model for 2- vs 3-photon isotropic chiral coherent control

In this case, the frequencies are such that *ω*_3_ = 2*ω*_2_ and *ω*_1_ + 2*ω*_2_ is resonant with the transition from the ground state $\left\vert g\right\rangle$ to the excited state $\left\vert e\right\rangle$. This leads to six sets of excitation pathways: two 2-photon pathways, $\left\vert g\right\rangle {\to}^ {{\omega }_{3}}\left\vert m\right\rangle {\to}^ {{\omega }_{1}}\left\vert e\right\rangle$ and $\left\vert g\right\rangle {\to}^ {{\omega }_{1}}\left\vert m\right\rangle {\to}^ {{\omega }_{3}}\left\vert e\right\rangle$, and three 3-photon pathways $\left\vert g\right\rangle {\to}^ {{\omega }_{2}}\left\vert n\right\rangle {\to}^ {{\omega }_{2}}\left\vert l\right\rangle {\to}^ {{\omega }_{1}}\left\vert e\right\rangle$, $\left\vert g\right\rangle {\to}^ {{\omega }_{2}}\left\vert n\right\rangle {\to}^ {{\omega }_{1}}\left\vert l\right\rangle {\to}^ {{\omega }_{2}}\left\vert e\right\rangle$, and $\left\vert g\right\rangle {\to}^ {{\omega }_{1}}\left\vert n\right\rangle {\to}^ {{\omega }_{2}}\left\vert l\right\rangle {\to}^ {{\omega }_{2}}\left\vert e\right\rangle$, where $\left\vert m\right\rangle$, $\left\vert n\right\rangle$, and $\left\vert l\right\rangle$ are intermediate states that have to be summed over. The probability amplitude for state $\left\vert e\right\rangle$ after the interaction is given by

17 $${a}_{e}=\mathop{\sum }\limits_{m}{a}_{e,m}+\mathop{\sum }\limits_{nl}{a}_{e,nl},$$

where perturbation theory yields

18 $${a}_{e,m}=\mathop{\sum }\limits_{\pi }{A}_{m}^{\pi \left({\omega }_{1},{\omega }_{3}\right)}\left({{{\bf{d}}}}_{e,m}\cdot {{{\bf{E}}}}_{1}^{\pi }\right)\left({{{\bf{d}}}}_{m,g}\cdot {{{\bf{E}}}}_{3}^{\pi }\right),$$

19 $${a}_{e,nl}=\mathop{\sum }\limits_{{\pi }^{{\prime} }}{A}_{nl}^{{\pi }^{{\prime} }\left({\omega }_{1},{\omega }_{2},{\omega }_{2}\right)}\left({{{\bf{d}}}}_{e,n}\cdot {{{\bf{E}}}}_{1}^{{\pi }^{{\prime} }}\right)\left({{{\bf{d}}}}_{n,l}\cdot {{{\bf{E}}}}_{2}^{{\pi }^{{\prime} }}\right)\left({{{\bf{d}}}}_{l,g}\cdot {{{\bf{E}}}}_{2}^{{\pi }^{{\prime} }}\right),$$

and the sums are over permutations π and ${\pi }^{{\prime} }$ of the photon orderings in each pathway. The interference between 2- and a 3-photon pathways will lead to an enantioselective contribution to the orientation-averaged population of the form

20 $$\frac{1}{2}\Delta {P}_{e}=	2{{\rm{Re}}}\left\{\mathop{\sum }\limits_{m,n,l}\mathop{\sum }\limits_{\pi,{\pi }^{{\prime} }}{A}_{m}^{\pi \left({\omega }_{1},{\omega }_{3}\right)*}{A}_{nl}^{{\pi }^{{\prime} }\left({\omega }_{1},{\omega }_{2},{\omega }_{2}\right)}\int{{\rm{d}}}\varrho {\left({{{\bf{d}}}}_{e,m}\cdot {{{\bf{E}}}}_{1}^{\pi }\right)}^{*}{\left({{{\bf{d}}}}_{m,g}\cdot {{{\bf{E}}}}_{3}^{\pi }\right)}^{*}\right.\\ 	\left({{{\bf{d}}}}_{e,n}\cdot {{{\bf{E}}}}_{1}^{{\pi }^{{\prime} }}\right)\left({{{\bf{d}}}}_{n,l}\cdot {{{\bf{E}}}}_{2}^{{\pi }^{{\prime} }}\right)\left({{{\bf{d}}}}_{l,g}\cdot {{{\bf{E}}}}_{2}^{{\pi }^{{\prime} }}\right)\bigg\}.$$

The integral over orientations can be performed analytically following ref. ^111^ (see e.g. the SI of ref. ^62^ for a worked out example). Each integral over orientations yields a result of the form **F** ⋅ *M***G**, where **F** (**G**) is a six-dimensional vector of rotational invariants involving triple products and dot products between electric field (electric dipole) vectors only, and *M* is a matrix coupling the molecular and the field invariants. See e.g. Eqs. (23)–(25). The different permutations in Eq. (20) will yield different **F**’s, which complicates interpretation. However, since a permutation of the electric fields **E**_*j*_ in the integral over orientations is equivalent to a corresponding permutation of the electric dipoles, we choose to keep the **E**_*j*_’s fixed and permute the dipoles instead. That is, we consider integrals with the form

21 $$\int{{\rm{d}}}\varrho {\left({{{\bf{d}}}}_{e,m}^{\pi }\cdot {{{\bf{E}}}}_{1}\right)}^{*}{\left({{{\bf{d}}}}_{m,g}^{\pi }\cdot {{{\bf{E}}}}_{3}\right)}^{*}\left({{{\bf{d}}}}_{e,n}^{{\pi }^{{\prime} }}\cdot {{{\bf{E}}}}_{1}\right)\left({{{\bf{d}}}}_{n,l}^{{\pi }^{{\prime} }}\cdot {{{\bf{E}}}}_{2}\right)\left({{{\bf{d}}}}_{l,g}^{{\pi }^{{\prime} }}\cdot {{{\bf{E}}}}_{2}\right),$$

where the permutations are now among the molecular dipoles. To simplify the expression for **F**, we reorder the dot products in the integral and apply Eq. (24) in ref. ^111^ according to

22 $$\int{{\rm{d}}}\varrho \left({{{\bf{d}}}}_{n,l}^{{\pi }^{{\prime} }}\cdot {{{\bf{E}}}}_{2}\right)\left({{{\bf{d}}}}_{l,g}^{{\pi }^{{\prime} }}\cdot {{{\bf{E}}}}_{2}\right)\left({{{\bf{d}}}}_{e,n}^{{\pi }^{{\prime} }}\cdot {{{\bf{E}}}}_{1}\right){\left({{{\bf{d}}}}_{e,m}^{\pi }\cdot {{{\bf{E}}}}_{1}\right)}^{*}{\left({{{\bf{d}}}}_{m,g}^{\pi }\cdot {{{\bf{E}}}}_{3}\right)}^{*}={{\bf{F}}}\cdot M{{{\bf{G}}}}_{\pi,{\pi }^{{\prime} }},$$

where

23 $${{\bf{F}}}=\left(\begin{array}{c}0\\ 0\\ 0\\ \left[{{{\bf{E}}}}_{2}\cdot ({{{\bf{E}}}}_{1}\times {{{\bf{E}}}}_{1}^{*})\right]({{{\bf{E}}}}_{2}\cdot {{{\bf{E}}}}_{3}^{*})\\ \left[{{{\bf{E}}}}_{2}\cdot ({{{\bf{E}}}}_{1}\times {{{\bf{E}}}}_{3}^{*})\right]({{{\bf{E}}}}_{2}\cdot {{{\bf{E}}}}_{1}^{*})\\ \left[{{{\bf{E}}}}_{2}\cdot ({{{\bf{E}}}}_{1}^{*}\times {{{\bf{E}}}}_{3}^{*})\right]({{{\bf{E}}}}_{2}\cdot {{{\bf{E}}}}_{1})\end{array}\right),$$

24 $$M=\frac{1}{30}\left(\begin{array}{cccccc}3&-1&-1&1&1&0\\ -1&3&-1&-1&0&1\\ -1&-1&3&0&-1&-1\\ 1&-1&0&3&-1&1\\ 1&0&-1&-1&3&-1\\ 0&1&-1&1&-1&3\end{array}\right),$$

25 $${{{\bf{G}}}}_{\pi,{\pi }^{{\prime} }}=\left(\begin{array}{c}\left[{{{\bf{d}}}}_{n,l}^{{\pi }^{{\prime} }}\cdot ({{{\bf{d}}}}_{l,g}^{{\pi }^{{\prime} }}\times {{{\bf{d}}}}_{e,n}^{{\pi }^{{\prime} }})\right]({{{\bf{d}}}}_{e,m}^{\pi }\cdot {{{\bf{d}}}}_{m,g}^{\pi })\\ \left[{{{\bf{d}}}}_{n,l}^{{\pi }^{{\prime} }}\cdot ({{{\bf{d}}}}_{l,g}^{{\pi }^{{\prime} }}\times {{{\bf{d}}}}_{e,m}^{\pi })\right]({{{\bf{d}}}}_{e,n}^{{\pi }^{{\prime} }}\cdot {{{\bf{d}}}}_{m,g}^{\pi })\\ \left[{{{\bf{d}}}}_{n,l}^{{\pi }^{{\prime} }}\cdot ({{{\bf{d}}}}_{l,g}^{{\pi }^{{\prime} }}\times {{{\bf{d}}}}_{m,g}^{\pi })\right]({{{\bf{d}}}}_{e,n}^{{\pi }^{{\prime} }}\cdot {{{\bf{d}}}}_{e,m}^{\pi })\\ \left[{{{\bf{d}}}}_{n,l}^{{\pi }^{{\prime} }}\cdot ({{{\bf{d}}}}_{e,n}^{{\pi }^{{\prime} }}\times {{{\bf{d}}}}_{e,m}^{\pi })\right]({{{\bf{d}}}}_{l,g}^{{\pi }^{{\prime} }}\cdot {{{\bf{d}}}}_{m,g}^{\pi })\\ \left[{{{\bf{d}}}}_{n,l}^{{\pi }^{{\prime} }}\cdot ({{{\bf{d}}}}_{e,n}^{{\pi }^{{\prime} }}\times {{{\bf{d}}}}_{m,g}^{\pi })\right]({{{\bf{d}}}}_{l,g}^{{\pi }^{{\prime} }}\cdot {{{\bf{d}}}}_{e,m}^{\pi })\\ \left[{{{\bf{d}}}}_{n,l}^{{\pi }^{{\prime} }}\cdot ({{{\bf{d}}}}_{e,m}^{{\pi }^{{\prime} }}\times {{{\bf{d}}}}_{m,g}^{\pi })\right]({{{\bf{d}}}}_{l,g}^{{\pi }^{{\prime} }}\cdot {{{\bf{d}}}}_{e,n}^{{\pi }^{{\prime} }})\end{array}\right).$$

Replacing in the expression for the enantiosensitive contribution to the population, we obtain

26 $$\frac{1}{2}\Delta {P}_{e}=2{{\rm{Re}}}\left\{{{\bf{F}}}\cdot M\mathop{\sum }\limits_{m,n,l}\mathop{\sum }\limits_{\pi,{\pi }^{{\prime} }}{A}_{m}^{\pi \left({\omega }_{1},{\omega }_{3}\right)*}{A}_{n,l}^{{\pi }^{{\prime} }\left({\omega }_{1},{\omega }_{2},{\omega }_{2}\right)}{{{\bf{G}}}}_{\pi,{\pi }^{{\prime} }}\right\}.$$

This reduces to Eqs. (3) and (4) after writing out the spatial oscillation of the field ${{{\bf{E}}}}_{j}({{\bf{r}}})={{{\bf{E}}}}_{j}({{{\bf{r}}}}_{0}){e}^{i{{{\bf{k}}}}_{j}\cdot ({{\bf{r}}}-{{{\bf{r}}}}_{0})}$ and defining **g**^(5)^ as

27 $${{{\bf{g}}}}^{\left(5\right)}\equiv 4{M}^{{\prime} }\mathop{\sum }\limits_{m,n,l}\mathop{\sum }\limits_{\pi,{\pi }^{{\prime} }}{A}_{m}^{\pi \left({\omega }_{1},{\omega }_{3}\right)*}{A}_{n,l}^{{\pi }^{{\prime} }\left({\omega }_{1},{\omega }_{2},{\omega }_{2}\right)}{{{\bf{G}}}}_{\pi,{\pi }^{{\prime} }},$$

where ${M}^{{\prime} }$ is the 3 × 6 matrix consisting of the 4th to 6th rows of *M*.

## Supplementary information

Transparent Peer Review file

## Data Availability

The geometries, energies, and transition dipoles of carvone, as well as the numerical data required to generate Figs. 2a, 3, and 4, have been made available in ref. ^113^.
