# Supplementary material for: Shaping light in 3D for direct asymmetric photochemistry: a 1000-fold enantioselectivity enhancement
Source: Nat Commun. 2026 Sep 4;17:9508. doi: 10.1038/s41467-026-77096-w (PMC13545228; doi:10.1038/s41467-026-77096-w)
Supplement: Supplementary file 1 [file 41467_2026_77096_MOESM1_ESM.pdf]

nature portfolio

Peer Review File

Shaping light in 3D for direct asymmetric photochemistry: a 1000-fold enantioselectivity enhancement

Corresponding Author: Dr Andrés Ordóñez

This file contains all reviewer reports in order by version, followed by all author rebuttals in order by version.

Version 0:

Reviewer comments:

Reviewer #1

(Remarks to the Author)
Ordonez et al. theoretically demonstrate that shaping multi-frequency laser fields so that their combined polarizations form a chiral Lissajous figure allows interference between multiphoton excitation pathways to selectively excite one molecular enantiomer over the other.
Using ab initio time-dependent simulations of randomly oriented racemic samples of carvone, they solve the time-dependent Schrödinger equation within the electric-dipole approximation and average over molecular orientations. These simulations show enantioselectivities of up to ~30%, around a thousand-fold enhancement compared to circularly polarised light.
The study further demonstrates two control schemes: one producing sub-wavelength chiral excitation patterns and another yielding uniform macroscopic enantioselectivity, showing that coherent phase and polarization control of tailored light fields can drive direct asymmetric photochemistry without chiral catalysts or enantiopure reagents.

The manuscript is clearly written and the results are very interesting. I can recommend publication after these minor issues being properly addressed.

1) The authors model gas-phase molecules using the fixed-nuclei approximation and averaging only over molecular orientations. This is a reasonable starting point for molecules in isolation. However, for this technique to be truly useful in practical photochemistry (particularly for asymmetric synthesis) it would need to operate in the condensed phase, where molecules experience thermal fluctuations, solvent interactions, and broader conformational ensembles. Do the authors expect their conclusions and the effectiveness of their proposed enantioselective excitation schemes to hold under such conditions? In particular, how might solvent effects, dynamic disorder, or vibrational averaging impact the observed enantioselectivity, especially given the subtle phase and polarization dependencies underlying the mechanism?

2) Does the presence of multiple closely spaced excited states represent a limitation of the proposed scheme? If so, could the authors specify for which classes of molecules (e.g., those with well-isolated electronic transitions) their approach is expected to be most effective?

3) It would be helpful if the authors could somehow evaluate how mismatches in the intensities of the ω, 2ω, and 3ω beams impact the observed enantioselectivity. This could also serve as a kind of error bar on their predictions for groups attempting to implement the scheme experimentally.

4) Color bar is missing in Figure 5

Reviewer #2

(Remarks to the Author)
Reviewer comments on the manuscript "Shaping light in 3D for direct asymmetric photochemistry: a 1000-fold enantioselectivity enhancement" by Ordóñez, Vindel-Zandbergen, and Ayuso.

This paper provides a computational and theoretical proposal for asymmetric photo-induced chemistry. Overall, this is nice work and I enjoyed learning about the work and results, although following the manuscript was a bit tedious. Generally, this looks like nice work and an interesting idea. It is directly based on a significant body of work and papers by the Smirnova group, which the first and last author significantly contributed to. To me, it looks like a interesting and potentially important application of that work in "real world chemistry" – but it doesn't really seem to live up to that, at least not in a way that I find this in the current manuscript.

Moreover, I have two central issues with the work: limited novelty/priority and limited clarity of the proposal.

The paper "Chiral templating of self-assembling nanostructures by circularly polarized light " [Nature Materials 14, 66 (2015)] demonstrates an "enantiomeric excess exceeding 30%" in the assembly of nanoscale structures.

Also, [34] in the manuscript demonstrates a 30% ee for separation of molecular ensembles, which should directly translate to the corresponding ee of any subsequent enantiospecific reaction, in the same way or even more general to the selective excitation in the current proposal.

There could be more related work that demonstrates ee >= 30 % resulting in corresponding enantioselective enhancement of chemical reactions and these should be looked up and considered.

Overall, to me this previous work limits the novelty of the work and it might be more appropriate to publish in a more specialized journal.

Second, if this is to be published in a general audience journal like Nat. Comm., the structure of the manuscript should be significantly improved. It should provide a clear description of the "proposal" or "computational demonstration" nature and should present a clear single proposal. Why does one need the "3 photon" model in the manuscript at all if it has very clear shortcomings whereas according to the authors the "5 photon" approach is the one that could possibly really be utilized? I would suggest to completely remove the "3 photon" model from the (main) manuscript in favor of structure and clarity of the paper.

Here are some more specific questions and comments that might help improving the manuscript:

Abstract: "standard chiral light" can influence chemical reactions when applied to molecules fixed in space [e.g., J. Chem. Phys. 154, 071101 (2021)].

In the second paragraph, it could already be pointed out in the current context that light can also serve as a catalyst, in light of [34] and the current work.

It would be appropriate to cite [Nature 497, 475–477 (2013)]. Could be brought up with respect to "Symmetry dictates (see Methods) that the total population transfer resulting from the interference ..." or earlier. (But not in the almost useless "[30-41]" statement.)

Regarding "liquid micro-jet of sub-wavelength thickness" this reviewer wonders if this could by any means be possible for UV/VIS excitations. How much "sub"-wavelength would one have to be for a realistic effect and is it possible/feasible to create liquid jets, e.g., of aqueous solutions, of such small thickness?

In line 157, if I understand correctly "choosing $\omega 1$ circularly polarised and $\omega 2$ and $\omega 3$ linearly polarised perpendicular to the plane of circular polarisation" says that the photon beams have to be perpendicular to each other, i.e., at least, the first one needs to be perpendicular to the others. This results in a very small volume and thus poses a severe limitation in general-chemistry approaches again. Should be clarified, clearly stated, and taken into account in

the claim toward general applicability.

Line 165, correctly, provides a reference to liquid-phase coherent control. But the authors should carefully consider why there is so little experimental evidence of "coherent control" in the liquid phase after the promising early work by Gerber and the promises they made back then. Moreover, the authors should discuss in how far inherently strong dephasing and decoherence processes (after excitation) in the condensed phase limits the ee of the actual chemical reactions in their proposed scheme.

Regarding the proposal of using "liquid microjet technology" I am wondering how this, in practice, would scale regarding sample consumption and overall efficiency of the process with respect to overall products and overall ee.

Regarding the simulations, one is left wondering in how far a "fixed nuclei approximation" is appropriate for computations related to chemical reactions. For instance, but not limited to, the "dephasing and decoherence" asked about above?

Version 1:

Reviewer comments:

Reviewer #1

(Remarks to the Author)
The authors have addressed all the suggested points, and the manuscript has improved considerably. I can now recommend it for publication.

Reviewer #2

(Remarks to the Author)
Reviewer comments on the manuscript "Shaping light in 3D for direct asymmetric photochemistry: a 1000-fold enantioselectivity enhancement" by Ordóñez, González, Vindel-Zandbergen, and Ayuso.

First, I note that there is an additional author on the updated manuscript.

Overall, I appreciate the authors' very careful and detailed response to both previous reviews and the corresponding updates to the manuscript, which resolved most questions and, at least, clearly addressed the few other ones such that the reader can decide themselves. This also helped clarifying several points for me. I am also happy that the authors kept the manuscripts length by simplifying and shortening the previous content/discussion before/while adding new details and explanations.

Generally, I am still somewhat pessimistic of the broad and general applicability with the promised selectivity, but I do see the rigor in the computational modeling/proposal and experimentalists (and time) will have to live up to it or bring forward the actual problems – we'll see:-)

I support publishing the updated current manuscript in Nat. Comm.

Open Access This Peer Review File is licensed under a Creative Commons Attribution 4.0 International License, which permits use, sharing, adaptation, distribution and reproduction in any medium or format, as long as you give appropriate credit to the original author(s) and the source, provide a link to the Creative Commons license, and indicate if changes were made.
In cases where reviewers are anonymous, credit should be given to 'Anonymous Referee' and the source.
The images or other third party material in this Peer Review File are included in the article's Creative Commons license, unless indicated otherwise in a credit line to the material. If material is not included in the article's Creative Commons license and your intended use is not permitted by statutory regulation or exceeds the permitted use, you will need to obtain permission directly from the copyright holder.
To view a copy of this license, visit https://creativecommons.org/licenses/by/4.0/

Reviewer #1 (Remarks to the Author):

Ordonez et al. theoretically demonstrate that shaping multi-frequency laser fields so that their combined polarizations form a chiral Lissajous figure allows interference between multiphoton excitation pathways to selectively excite one molecular enantiomer over the other. Using ab initio time-dependent simulations of randomly oriented racemic samples of carvone, they solve the time-dependent Schrödinger equation within the electric-dipole approximation and average over molecular orientations. These simulations show enantioselectivities of up to ~30%, around a thousand-fold enhancement compared to circularly polarised light. The study further demonstrates two control schemes: one producing sub-wavelength chiral excitation patterns and another yielding uniform macroscopic enantioselectivity, showing that coherent phase and polarization control of tailored light fields can drive direct asymmetric photochemistry without chiral catalysts or enantiopure reagents.

The manuscript is clearly written and the results are very interesting. I can recommend publication after these minor issues being properly addressed.

We thank the referee for their very positive comments.

1) The authors model gas-phase molecules using the fixed-nuclei approximation and averaging only over molecular orientations. This is a reasonable starting point for molecules in isolation. However, for this technique to be truly useful in practical photochemistry (particularly for asymmetric synthesis) it would need to operate in the condensed phase, where molecules experience thermal fluctuations, solvent interactions, and broader conformational ensembles. Do the authors expect their conclusions and the effectiveness of their proposed enantioselective excitation schemes to hold under such conditions? In particular, how might solvent effects, dynamic disorder, or vibrational averaging impact the observed enantioselectivity, especially given the subtle phase and polarization dependencies underlying the mechanism?

Yes, we expect our technique to remain effective in the liquid phase. First, let us highlight that our scheme relies on instantaneous interference with both pathways acting simultaneously and does not rely on wave packet dynamics. This is also the case for standard electronic circular dichroism (CD), which relies on interference between electric-dipole and magnetic-dipole transitions, and which is routinely measured in liquids. Instantaneous interference between 2-photon pathways has also been successfully applied for coherent control in liquids in several experiments (see Refs. [69-80] in the updated manuscript). Thus, the interference as such is not an issue in liquids. We have added the following text to clarify this:

"Coherent control of electronic population in polyatomic molecules has been experimentally demonstrated in gas and liquid samples [67,68]. In the liquid phase, interference between simultaneously driven 2-photon pathways in the absence of intermediate resonances has proven to be a robust approach to controlling population transfer [69–80]. Our approach relies on tailoring a light field using three frequencies, which may be an intense IR source and two of its harmonics, to simultaneously drive a 2- and a 3-photon pathway, in the absence of intermediate resonances, without

resorting to excited wave packet dynamics, and requiring only a stable relative phase between the two co-propagating frequencies."

The liquid environment will broaden and shift the molecular spectrum in a solvent-dependent way and the wavelength in our scheme must be adjusted accordingly. This is also analogous to what occurs with CD and to the liquid-phase coherent control experiments mentioned above.

As the timescale of the electronic response (attoseconds) is much shorter than the timescale of the dynamic disorder in a liquid (tens to hundreds of femtoseconds), its effect is equivalent to averaging the response over many possible geometries. We have investigated how fluctuations of the molecular geometry affect the enantioselectivity of our scheme. We found that some geometries simply fall out of resonance and are not significantly excited, while the ones that remain in resonance display varying degrees of enantioselectivity. Crucially, these keep the same sign of enantioselectivity and thus integrating over geometries still leads to a high enantioselectivity ~19%. We expect that larger fluctuations of the molecular geometry will be out of resonance and thus will not contribute significantly to the integral over geometries. We have added a figure showing these results (Fig. 4c) and the following text to the updated manuscript.

"To address robustness with respect to molecular parameters, we calculated the coherent control curve $\gamma(\Delta\phi)$ for 100 molecular geometries randomly sampled from a Wigner distribution of the vibrational ground state of carvone (see Fig. 4c), while keeping all field parameters fixed to their values in Fig. 3. Of the 100 sampled geometries, 45 remain in near 5ω-resonance with the applied field and are thus significantly populated compared to the equilibrium geometry, as indicated by the colour scale in Fig. 4c. Although the amplitude of the individual $\gamma(\Delta\phi)$ curves is very sensitive to their molecular geometry, their phase is largely preserved, and thus the effect survives ensemble averaging. Specifically, we still obtain a high $\gamma \approx 19\%$ (see thick black curve in Fig. 4c) when we compute it using $P_{S/R} = \sum_i P_{S/R}^{(i)}$, where $P_{S/R}^{(i)}$ is the population for the i-th geometry in the R/S enantiomer and the sum runs over all configurations. This shows that the enantioselective response remains robust under averaging over the ground-state nuclear distribution, reflecting the fact that all geometries share the same underlying molecular chirality. We have not attempted an optimization of the total enantioselectivity, thus $\gamma \approx 19\%$ is a lower bound for the optimal total enantioselectivity"

2) Does the presence of multiple closely spaced excited states represent a limitation of the proposed scheme? If so, could the authors specify for which classes of molecules (e.g., those with well-isolated electronic transitions) their approach is expected to be most effective?

From a fundamental point of view, the molecule will experience a strongly chiral interaction with the field (occurring within the electric-dipole approximation) regardless of the details of its electronic structure. Thus, we do not expect our scheme to be fundamentally limited to molecules with a well-isolated excited electronic state, such as carvone. Nevertheless, when several closely spaced excited states are simultaneously populated, the enantioselectivity will generally vary from state to state.

Thus, the enantioselectivity averaged over the populated states may potentially suffer from partial cancellations. Whether the amount of cancellation is significant remains to be investigated. But if significant cancellation occurs, it may still be possible to mitigate it by exploring the vast parameter space provided by the different polarization combinations, as dictated by Eq. (3). Indeed, even if the states are close in energy, their dipole couplings to other states may be considerably different, leading to a different $\vec{g}^{(5)}$, which can be then addressed by changing $\vec{h}^{(5)}$ via the polarizations [Eqs. (3) and (4)]. We have added the following sentence in the conclusions:

"Similarly, in situations where the target state is nearly energy-degenerate with other excited states and enantioselectivity may be reduced due to state averaging, the vast parameter space offered by the different possible polarization combinations may offer a compromise between state selectivity and high enantioselectivity."

3) It would be helpful if the authors could somehow evaluate how mismatches in the intensities of the ω, 2ω, and 3ω beams impact the observed enantioselectivity. This could also serve as a kind of error bar on their predictions for groups attempting to implement the scheme experimentally.

We thank the referee for this thoughtful suggestion. We have performed a systematic study of the enantioselectivity as a function of the field intensities. Our findings are summarized in Figs. 4a and b in the new manuscript and in the following text:

"To address the robustness of our approach with respect to variations in the intensity of the driving fields and molecular geometry, we calculated the enantioselectivity γ at $\Delta\phi = 0$ for a wide range of intensity combinations, as shown in Figures 4a and b. For a fixed value of I_{ω}, one can see that high enantioselectivity requires a balance between $I_{2\omega}$ and $I_{4\omega}$, which control the weight of each pathway (see Fig. 2b). As can be seen in Figs. 4a and b, this balance can be maintained over a broad region of intensities. Moreover, since one ω photon enters each pathway, the balance between the two pathways is largely independent of I_{ω}, as can be seen from the similarity between Figs. 4a and b, which correspond to values of I_{ω} that differ from each other by over an order of magnitude."

4) Color bar is missing in Figure 5

Thank you for pointing this out, we have added the missing colorbar (Fig. 3 in the updated manuscript).

Reviewer #2 (Remarks to the Author):

Reviewer comments on the manuscript "Shaping light in 3D for direct asymmetric photochemistry: a 1000-fold enantioselectivity enhancement" by Ordóñez,

Vindel-Zandbergen, and Ayuso.

This paper provides a computational and theoretical proposal for asymmetric photo-induced chemistry. Overall, this is nice work and I enjoyed learning about the work and results, although following the manuscript was a bit tedious. Generally, this looks like nice work and an interesting idea. It is directly based on a significant body of work and papers by the Smirnova group, which the first and last author significantly contributed to. To me, it looks like a interesting and potentially important application of that work in "real world chemistry" – but it doesn't really seem to live up to that, at least not in a way that I find this in the current manuscript.

Moreover, I have two central issues with the work: limited novelty/priority and limited clarity of the proposal.

We thank the referee for their positive comments about work, for considering it an interesting and potentially important application to real-world chemistry, and for their constructive criticism, which has helped us improve the manuscript.

Following the referee's comments and suggestions, we have substantially modified the manuscript to improve its clarity. In the revised version, we highlight the novelty of our work by emphasising the key fundamental differences from previous related works, including those in the references provided by the referee. All the changes, described below, are highlighted in blue in the updated manuscript.

The paper "Chiral templating of self-assembling nanostructures by circularly polarized light " [Nature Materials 14, 66 (2015)] demonstrates an "enantiomeric excess exceeding 30%" in the assembly of nanoscale structures.

Ref. [Nature Materials 14, 66 (2015)] studies light-induced ***self-assembly*** *of* ***metallic nanoribbons (size ~1μm)***, which occur on a timescale of over ***tens of hours*** and is driven by ***standard circularly polarized light*** via ***linear light-matter interactions***.

Our work addresses ***enantioselective electronic excitation*** of ***organic molecules (size ~1 nm)*** on ***ultrafast timescales (tens of fs)*** driven by ***3D-shaped light*** via ***nonlinear*** and ***purely electric-dipole interactions***.

Thus, our work is fundamentally very different from Ref. [Nature Materials 14, 66 (2015)]:

- ***Different physical systems:*** metallic nanoribbons vs organic molecules
- ***Different phenomena***: self-assembly vs electronic population transfer
- ***Different timescales***: tens of hours vs femtoseconds
- ***Different spatial scales***: ~1μm vs ~1nm
- ***Different regime of light-matter interactions***: linear vs nonlinear. Note that the linear effects responsible for the observations in [Nature Materials 14, 66 (2015)] are negligible (<0.01%) in our work—our approach relies exclusively on nonlinear effects driven by purely electric-dipole interactions.

- ***Different type of optical chirality*:** standard circularly polarized light vs 3D-shaped light. Note that standard chiral light (used in [Nature Materials 14, 66 (2015)]) cannot fundamentally drive enantioselective population transfer in randomly oriented chiral molecules via purely electric-dipole interactions. This requires shaping light in 3D, as we demonstrate in our paper.
- ***Different potential applications:*** the authors of [Nature Materials 14, 66 (2015)]) see "the synthesis of chiral ***photonic materials***" as the main potential application of their work, whereas in our work it is the synthesis and manipulation of chiral ***molecules***.

To avoid any possible misunderstandings, we cited [Nature Materials 14, 66 (2015)] (new Ref. [40]) in the updated manuscript and added the following text:

"Indeed, while $e.e. \sim 30\%$ has been achieved in the self-assembly of nanoparticles [16, 40], direct asymmetric photochemistry involving small- to medium-size organic molecules has remained barely enantioselective."

Also, [34] in the manuscript demonstrates a 30% ee for separation of molecular ensembles, which should directly translate to the corresponding ee of any subsequent enantiospecific reaction, in the same way or even more general to the selective excitation in the current proposal.

Ref. [34] ([39] in the updated manuscript) presents a theoretical proposal for separating chiral molecules in the ***gas phase*** via ***rotational excitation***, which requires ***ultracold samples*** (<1K). Therefore, it is ***not applicable to liquids or chemical synthesis***. Indeed, rotational excitation does not trigger photochemical reactions at all.

Our paper addresses enantioselective ***electronic excitation triggering photochemical reactions***. In contrast to previous rotational-based proposals, such as [34] ([39]), our approach is applicable to ***chemically relevant liquid samples*** and works at ***room temperature.***

This makes our work fundamentally different to any previous works based on rotational excitation, including Ref. [34] ([39]):

- ***Different phenomena:*** rotational vs electronic excitation
- ***Different states of matter***: gas vs liquid phase
- ***Different requirements***: Ref. [34] ([39]) requires ultracold samples whereas our approach works at room temperature
- ***Different timescales***: picoseconds vs femtoseconds
- ***Different regime of light-matter interactions***: rotational excitation uses microwave radiation whereas electronic excitation requires optical fields. Let us emphasise that the different scale of the wavelengths used (cm vs μm) has long posed a fundamental challenge for electronic excitation. Our paper introduces, for the first time, a route to overcome this challenge.
- ***Different potential applications:*** in contrast with our work based on electronic excitation, approaches relying on rotational excitation, such as [34] ([39]), are not applicable to liquids or chemical synthesis.

We have moved the citation to [34] ([39]) earlier in the introduction, together with the following text, to avoid any possible misunderstandings:

"... novel approaches relying exclusively on electric-dipole interactions enable enhanced chiral sensitivity without suppressing the overall response [31]. These include chiral sum-frequency generation [32], Coulomb explosion imaging [33, 34], photoelectron circular dichroism [35], as well as enantioselective detection [36] and manipulation via microwave rotational excitation [37, 38], which could lead to enantioseparation [39]."

There could be more related work that demonstrates ee >= 30 % resulting in corresponding enantioselective enhancement of chemical reactions and these should be looked up and considered.

To the best of our knowledge, we have cited all the works relevant to the subject of our manuscript. If the referee has any concrete reference in mind, we will be more than happy to discuss including it, as we did regarding the references to the works on rotational excitation and nanoribbon self-assembly. We remark that our objective is not simply to achieve ee>30% but to do it in a way that works for molecules, is relevant to photochemistry, and is scalable to liquids.

Overall, to me this previous work limits the novelty of the work and it might be more appropriate to publish in a more specialized journal.

As described above, these previous works differ from our work in several fundamental ways. Most importantly, neither of them deals with enantioselective electronic excitation of chiral molecules, which is the core of our work. Furthermore, these works offer no prospect of enantioselective photochemistry at the molecular level. [Nature Materials 14, 66 (2015)] (Ref. [40] in the updated manuscript) deals with nanoparticles, and Ref. [34] (Ref. [39] in the updated manuscript) with rotational transitions (see detailed explanations above).

We have edited the introduction of the manuscript as described above to clarify the many fundamental differences between these works and our work.

Second, if this is to be published in a general audience journal like Nat. Comm., the structure of the manuscript should be significantly improved. It should provide a clear description of the "proposal" or "computational demonstration" nature and should present a clear single proposal. Why does one need the "3 photon" model in the manuscript at all if it has very clear shortcomings whereas according to the authors the "5 photon" approach is the one that could possibly really be utilized? I would suggest to completely remove the "3 photon" model from the (main) manuscript in favor of structure and clarity of the paper.

Following the referee's suggestion, we have re-written the paper to improve its clarity. In the new version, we have removed most of the 1-vs-2-photon approach, explaining only why its enantioselectivity oscillates rapidly in space and a 2-vs-3-photon approach is necessary. The paper now focuses on our strongest result: the 2-vs-3-photon approach.

Here are some more specific questions and comments that might help improving the manuscript:

Abstract: "standard chiral light" can influence chemical reactions when applied to molecules fixed in space [e.g., J. Chem. Phys. 154, 071101 (2021)].

Following the referee's suggestion, we have added citation to this work in the introduction (Ref. [34] in the updated manuscript). We believe that citing this work in the introduction (rather than at the beginning of the abstract) is more appropriate due to the several fundamental differences with our work described in the following, as otherwise it may confuse the reader.

We note that this paper, titled "Detecting handedness of spatially oriented molecules by Coulomb explosion imaging", by the group of J. Küpper, discriminates between opposite molecular enantiomers by recording the distributions of molecular fragments after Coulomb explosion of aligned molecules. In contrast with our work, this work focuses on post-selected aligned molecules (we consider randomly oriented samples) in the gas phase (we consider the liquid phase), uses Coulomb explosion imaging, and it does not address enantioselective excitation of electronic states or direct asymmetric photochemistry.

In the second paragraph, it could already be pointed out in the current context that light can also serve as a catalyst, in light of [34] and the current work.

In the second paragraph, we discuss catalysis in the context of asymmetric synthesis and photochemistry. A defining property of a catalyst is that it is not consumed in the reaction. Both in [34] ([39] in the updated manuscript) and in our work, photons are consumed in the excitation process, thus behaving as reactants rather than catalysts. Therefore, calling light a catalyst would be technically incorrect. Furthermore, photochemistry relies on electronic excitation and [34] ([39]) is about rotational excitation.

For these reasons, we believe that adding citation to Ref. [34] ([39]) in the fourth paragraph of the introduction (as described above), is more adequate than in this part of the introduction.

It would be appropriate to cite [Nature 497, 475–477 (2013)]. Could be brought up with respect to "Symmetry dictates (see Methods) that the total population transfer resulting from the interference ..." or earlier. (But not in the almost useless "[30-41]" statement.)

Following the referee's suggestion, we have added citation to this paper (Ref. [36] in the updated manuscript) and to other relevant works earlier in the introduction (fourth paragraph), together with a sentence that describes them.

We note that this paper, titled "*Enantiomer-specific **detection** of chiral molecules via microwave spectroscopy*", is about detecting opposite chiral molecules by measuring the phase of the emitted microwave radiation due to rotational dynamics. The populations induced in this work are the same in both enantiomers. Thus, we prefer

citing this reference earlier in the introduction rather than in connection to enantioselective population transfer, as otherwise it could confuse the reader.

Regarding "liquid micro-jet of sub-wavelength thickness" this reviewer wonders if this could by any means be possible for UV/VIS excitations. How much "sub"-wavelength would one have to be for a realistic effect and is it possible/feasible to create liquid jets, e.g., of aqueous solutions, of such small thickness?

This comment refers to fundamental wavelength in the 1-vs-2-photon setup, which has been removed in the last version of the manuscript following the referee's suggestion. The 2-vs-3-photon setup (our strong result) does not have this requirement. In any case, we are more than happy to answer the referee's question.

In the results presented in the previous version of the manuscript (now removed), we considered the IR fundamental wavelength λ (956 nm in vacuum, 720 nm in water), which is the one that determines the variation of the enantioselectivity. [Nat Comm 9, 1353 (2018)] (Ref. [90] in the updated manuscript) reports water flat liquid jet thickness as low as ~20 nm. More recent examples with sub 100 nm thickness can be found e.g. in [PCCP 27, 6457 (2025) and Rev. Sci. Instrum. 96, 073002 (2025)] (Refs. [94, 95] in the updated manuscript). This is well below 720 nm and therefore largely avoids the washing out of enantioselectivity.

In line 157, if I understand correctly "choosing ω1 circularly polarised and ω2 and ω3 linearly polarised perpendicular to the plane of circular polarisation" says that the photon beams have to be perpendicular to each other, i.e., at least, the first one needs to be perpendicular to the others. This results in a very small volume and thus poses a severe limitation in general-chemistry approaches again. Should be clarified, clearly stated, and taken into account in the claim toward general applicability. *

The referee is correct in recognizing that (in a thick transparent medium) the volume of the effective interaction region decreases with increasing angle between the beams. However, there are several reasons why we do not consider this to be a severe limitation:

1. In liquid-phase photochemistry, due to Beer-Lambert's law, the penetration depth (instead of the Rayleigh length) can often be the factor limiting the effective volume. Indeed, photochemistry is often done on thin (<1 mm) samples (see e.g. [Photochem Photobiol Sci 7, 1313 (2008)], [Tetrahedron Letters 53 (2012) 1363]). For beam waists on the same order of the thickness of the sample, crossing the beams at large angles does not reduce the effective volume significantly.

2. The intensities required are not so high that they require tight focusing. Thus, crossed beam experiments can be done with loose focusing (large beam waists), which naturally enhances the overlap volume.

In the updated manuscript we added:

"Furthermore, the integration of liquid microjet technology in femtosecond laser setups [90-95] facilitates multiphoton experiments by providing a continuously refreshed, thin sample that minimizes optical scattering, dispersion, and thermal effects. *For such thin*

samples, a large angle between the non-collinear beams does not significantly reduce the volume of the interaction region"

Line 165, correctly, provides a reference to liquid-phase coherent control. But the authors should carefully consider why there is so little experimental evidence of "coherent control" in the liquid phase after the promising early work by Gerber and the promises they made back then. Moreover, the authors should discuss in how far inherently strong dephasing and decoherence processes (after excitation) in the condensed phase limits the ee of the actual chemical reactions in their proposed scheme.

We consider there is enough evidence of multiphoton coherent control of population transfer in the liquid phase. We have added several new references to support this claim (Refs. [69-80] in the updated manuscript). These experiments rely on the **instantaneous** interference between several two-photon pathways driven by a broadband pulse to achieve selective two-photon excitation. It works because, like our approach, it does not require coherence between electronic states for periods of time exceeding the typical dephasing and decoherence times in liquids. <u>Note that standard electronic circular dichroism is the result of instantaneous interference between the electric and magnetic dipole interactions, and it is routinely measured in liquids.</u>

Interferences are not per se fragile in a liquid environment—electronic wave packets are fragile, but our approach does not rely on them. Our approach relies on selectively driving population to an electronic excited state. After populating it, the molecule isomerizes as usual, i.e. without relying on quantum interferences. The amount of isomerization is given by the quantum yield Y (which is not enantioselective). Thus, the enantioselectivity in the production of the photoproduct (e.g. carvonecamphor) will be $2(YP_R - YP_S)/(YP_R + YP_S) = 2(P_R - P_S)/(P_R + P_S)$, which is independent of the quantum yield Y and the same as for the excited state population.

We have clarified this in the updated manuscript as follows:

"Coherent control of electronic population in polyatomic molecules has been experimentally demonstrated in gas and liquid samples [67, 68]. In particular, in the liquid phase, interference between simultaneously driven 2-photon pathways in the absence of intermediate resonances has proven to be a robust approach to controlling population transfer [69–80]. Our approach relies on tailoring a light field using three frequencies, which may be an intense IR source and two of its harmonics, to simultaneously drive a 2- and a 3-photon pathway, in the absence of intermediate resonances, without resorting to excited wave packet dynamics, and requiring only a stable relative phase between the two co-propagating frequencies."

Regarding the proposal of using "liquid microjet technology" I am wondering how this, in practice, would scale regarding sample consumption and overall efficiency of the process with respect to overall products and overall ee.

When using a liquid microjet, the sample can be recycled and pass many times through the jet nozzle, as it is standard in experiments with liquid microjets. We have added the following sentence to the manuscript:

"Moreover, the sample can be recycled and pass multiple times through the interaction region."

Regarding the simulations, one is left wondering in how far a "fixed nuclei approximation" is appropriate for computations related to chemical reactions. For instance, but not limited to, the "dephasing and decoherence" asked about above?

The chemical reaction occurs after the photoexcitation event. Since it is well known that carvone isomerizes upon excitation [54-59], we focus on the excitation step. The photoexcitation timescale is determined by the electronic response, which occurs on the attosecond timescale. The nuclei can be safely considered fixed on such timescale. Nevertheless, related to this question, we performed further calculations to estimate the effect of a spread of nuclear geometries, and summarized our findings in the following addition to the manuscript:

"To address robustness with respect to molecular parameters, we calculated the coherent control curve $\gamma(\Delta\phi)$ for 100 molecular geometries randomly sampled from a Wigner distribution of the vibrational ground state of carvone (see Fig. 4c), while keeping all field parameters fixed to their values in Fig. 3. Of the 100 sampled geometries, 45 remain in near 5ω-resonance with the applied field and are thus significantly populated compared to the equilibrium geometry, as indicated by the colour scale in Fig. 4c. Although the amplitude of the individual $\gamma(\Delta\phi)$ curves is very sensitive to their molecular geometry, their phase is largely preserved, and thus the effect survives ensemble averaging. Specifically, we still obtain a high $\gamma \approx 19\%$ (see thick black curve in Fig. 4c) when we compute it using $P_{S/R} = \sum_i P_{S/R}^{(i)}$, where $P_{S/R}^{(i)}$ is the population for the i-th geometry in the R/S enantiomer and the sum runs over all configurations. This shows that the enantioselective response remains robust under averaging over the ground-state nuclear distribution, reflecting the fact that all geometries share the same underlying molecular chirality. We have not attempted an optimization of the total enantioselectivity, thus $\gamma \approx 19\%$ is a lower bound for the optimal total enantioselectivity "
